# Long-term exposure to ambient ozone at workplace is positively and non-linearly associated with incident hypertension and blood pressure: longitudinal evidence from the Beijing-Tianjin-Hebei medical examination cohort

**DOI:** 10.1186/s12889-023-16932-w

**Published:** 2023-10-16

**Authors:** Songhua Hu, Ximing Xu, Chunjun Li, Li Zhang, Xiaolong Xing, Jiangshan He, Pei Guo, Jingbo Zhang, Yujie Niu, Shuo Chen, Rong Zhang, Feng Liu, Shitao Ma, Mianzhi Zhang, Fenghua Guo, Minying Zhang

**Affiliations:** 1 School of Statistics and Data Science, Nankai University, Tianjin, China; 2 Big Data Center for Children’s Medical Care, Children’s Hospital of Chongqing Medical University, National Clinical Research Center for Child Health and Disorders, Ministry of Education Key Laboratory of Child Development and Disorders, Chongqing, China; 3 Tianjin Union Medical Center, Tianjin, China; 4 Tianjin First Central Hospital, Tianjin, China; 5 School of Medicine, Nankai University, Tianjin, China; 6 Beijing Physical Examination Center, Beijing, China; 7 Hebei Key Laboratory of Environment and Human Health, Shijiazhuang, China; 8 Department of Occupational Health and Environmental Health, Hebei Medical University, Shijiazhuang, China; 9 Dongfang Hospital, Beijing University of Chinese Medicine, Beijing, China; 10 Tianjin Academy of Traditional Chinese Medicine Affiliated Hospital, Tianjin, China

**Keywords:** Air pollution, Ozone, Hypertension, Blood pressure, Occupational exposure, Longitudinal studies

## Abstract

**Background:**

There is limited longitudinal evidence on the hypertensive effects of long-term exposure to ambient O_3_. We investigated the association between long-term O_3_ exposure at workplace and incident hypertension, diastolic blood pressure (DBP), systolic blood pressure (SBP), pulse pressure (PP), and mean arterial pressure (MAP) in general working adults.

**Methods:**

We conducted a cohort study by recruiting over 30,000 medical examination attendees through multistage stratified cluster sampling. Participants completed a standard questionnaire and comprehensive medical examination. Three-year ambient O_3_ concentrations at each employed participant’s workplace were estimated using a two-stage machine learning model. Mixed-effects Cox proportional hazards models and linear mixed-effects models were used to examine the effect of O_3_ concentrations on incident hypertension and blood pressure parameters, respectively. Generalized additive mixed models were used to explore non-linear concentration-response relationships.

**Results:**

A total of 16,630 hypertension-free working participants at baseline finished the follow-up. The mean (SD) O_3_ exposure was 45.26 (2.70) ppb. The cumulative incidence of hypertension was 7.11 (95% CI: 6.76, 7.47) per 100 person-years. Long-term O_3_ exposure was independently, positively and non-linearly associated with incident hypertension (Hazard ratios (95% CI) for Q2, Q3, and Q4 were 1.77 (1.34, 2.36), 2.06 (1.42, 3.00) and 3.43 (2.46, 4.79), respectively, as compared with the first quartile (Q1)), DBP (*β* (95% CI) was 0.65 (0.01, 1.30) for Q2, as compared to Q1), SBP (*β* (95% CI) was 2.88 (2.00, 3.77), 2.49 (1.36, 3.61) and 2.61 (1.64, 3.58) for Q2, Q3, and Q4, respectively), PP (*β* (95% CI) was 2.12 (1.36, 2.87), 2.03 (1.18, 2.87) and 2.14 (1.38, 2.90) for Q2, Q3, and Q4, respectively), and MAP (*β* (95% CI) was 1.39 (0.76, 2.02), 1.04 (0.24, 1.84) and 1.12 (0.43, 1.82) for Q2, Q3, and Q4, respectively). The associations were robust across sex, age, BMI, and when considering PM_2.5_ and NO_2_.

**Conclusions:**

To our knowledge, this is the first cohort study in the general population that demonstrates the non-linear hypertensive effects of long-term O_3_ exposure. The findings are particularly relevant for policymakers and researchers involved in ambient pollution and public health, supporting the integration of reduction of ambient O_3_ into public health interventions.

**Supplementary Information:**

The online version contains supplementary material available at 10.1186/s12889-023-16932-w.

## Introduction

Hypertension, with its increasing prevalence, has become one of the leading risk factors for the global disease burden [1]. In China, hypertension remains a critical public health issue with a high prevalence [2] and a low control rate [3]. In recent decades, a substantial number of population-based studies have suggested the causal role of ambient pollutants on the incidence and prevalent hypertension [4].

Ozone (O_3_) imposes huge challenges to public health in China and globally [5]. In recent years, as many countries have gradually intensified their efforts to control air pollution, the concentrations of particulate matter (PM) have been decreasing, whereas the ambient O_3_ concentrations have been stable or even gradually increasing at the global level [1, 6]. In the 74 key cities in China, between 2013 and 2017, the annual average concentration of particulate matter with an aerodynamic diameter ≤ 2.5 μm (PM_2.5_) decreased by 33.3%, while the annual average concentration of O_3_ increased by 20.4%, for instance, the average annual concentration of O_3_ in 2017 was 163.0 µg/m^3^, considerably higher than the target set by WHO global air quality guidelines (peak-season average for O_3_ concentration of 100 µg/m^3^ as interim target 1) [7]; the adverse health events attributed to O_3_ exposure had increased consequently [8].

Although the evidence for the hypertensive effects of ambient pollutants has been well established over the past few decades, of particular concern is PM_2.5_, which has been implicated as a major contributor to unfavorable health outcomes [4]. Studies on the health impact of O_3_ have mostly focused on respiratory disease [9, 10], while evidence for the hypertensive effects of O_3_ exposure remains scarce, especially for long-term exposure to O_3_. Moreover, the results of the association between long-term exposure to O_3_ and hypertension have been mixed, with some reporting positive associations [11, 12], some finding adverse associations [13], and others showing non-significant associations [14, 15]. Due to the very limited number of association studies between long-term O_3_ exposure and hypertension, even a systematic review could not reach a robust conclusion on the plausibility of its association with hypertension [4]. In addition, most of the existing studies are limited by their cross-sectional design [11–13, 15]. The only two cohort studies were both conducted in specific populations, African American population with a high (56%) prevalence of hypertension [14] and American black women [16]. Thus, the inconsistency of results from limited studies and the lack of longitudinal evidence warrant prospective cohort studies on the hypertensive effects of long-term O_3_ exposure, especially in the general population in typically polluted areas.

O_3_ is a secondary ambient pollutant. The genesis of O_3_ is intricately linked to meteorological conditions, with its formation favored by high temperatures, strong radiation, low humidity, and light wind. Consequently, it predominantly appears during late spring, summer, and fall, when the sky is typically clear and clouds are scarce [17]. The near-surface O_3_ concentrations generally follow a diurnal pattern [18, 19], starting low in the early morning and gradually escalating as sunlight facilitates the accumulation of O_3_ precursors. The concentrations peak between 2:00 and 5:00 p.m., and then slowly decline as solar radiation subsides, reaching lower concentrations in the evening [20]. Therefore, assessing O_3_ concentrations where people spend their daytime when concentrations are high in polluted regions provides a more accurate estimate of the health risks posed by O_3_ exposure. To our knowledge, the existing studies on the health risks of O_3_ have assessed participants’ O_3_ exposure levels based on their residential addresses [21, 22], this may have misestimated the O_3_ exposure of the working population who spend their daytime at workplace and biased the results. Therefore, estimating O_3_ exposure at their workplaces would yield a more accurate assessment of health risks for working population.

The current study was limited to employed adults free of hypertension and aimed to investigate the relationship between long-term exposure to O_3_ at workplace and incident hypertension, diastolic blood pressure (DBP), systolic blood pressure (SBP), pulse pressure (PP), and mean arterial pressure (MAP), with full consideration of ambient PM_2.5_, nitrogen dioxide (NO_2_), and individual-level risk factors, and to examine the concentration-response curves to fill the knowledge gap on the hypertensive effects associated with long-term exposure to O_3_ in the Beijing-Tianjin-Hebei (BTH) region, a highly polluted area with elevated prevalence of hypertension in China [23, 24].

## Methods

### Study population

The Beijing-Tianjin-Hebei Medical Examination-based Cohort (BTH-MEC) recruited individuals undergoing annual or bi-annual medical examinations by multistage stratified cluster sampling at six tertiary hospitals in the BTH area, China. The baseline survey was conducted from July 2017 to October 2020, and the first follow-up was completed by the end of 2021. The cohort consisted of over 30,000 adults who had completed a questionnaire and a comprehensive medical examination. A detailed description of the study design and population has been reported elsewhere [25].

**Fig. 1:**
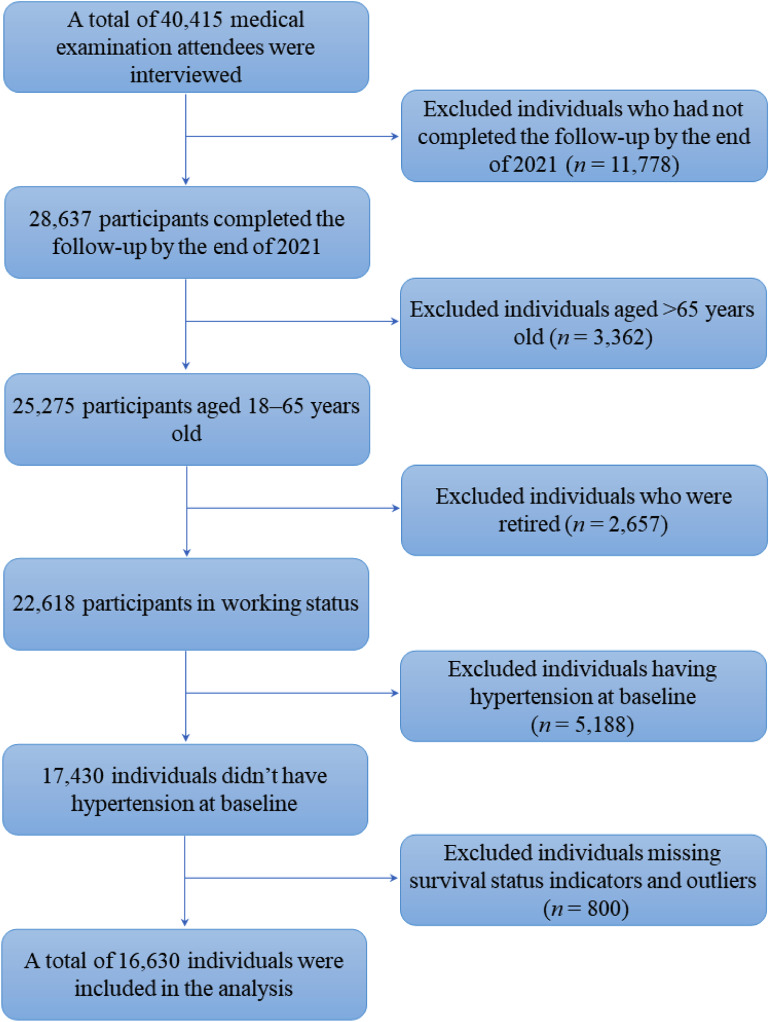
Flow chart of the inclusion and exclusion of participants in the BTH-MEC.

Among 28,637 participants who completed the follow-up, we excluded 5,988 individuals for the following reasons: being diagnosed with hypertension and/or using hypotensive drugs at baseline survey to eliminate the impact of hypotensive drugs on DBP or SBP (*n* = 5,188); missing blood pressure data (*n* = 800). Because we assessed the participants’ air pollution exposure based on the geographical locations of their workplaces, we additionally excluded retired individuals from the cohort, including those older than 65 years (*n* = 3,362) and/or those who were younger than 65 years but self-reported to be retired at the time of the interview (*n* = 2,657). The flow diagram for inclusion and exclusion is presented in Fig. 1.

### Air pollution exposure measures

Daily ambient O_3_ concentrations were estimated at a spatial resolution of 0.1° × 0.1° (latitude by longitude) using a two-stage machine learning model with multi-source data, including ground-based O_3_ monitoring data, satellite-derived aerosol optical depth (AOD), satellite ancillary covariates, meteorological variables, land use information, Weather Research and Forecasting (WRF) and Community Multi-scale Air Quality (CMAQ) simulated data, population distribution, and other ancillary variables [26]. The individual’s O_3_ exposure was estimated by assigning the predicted O_3_ concentrations to each participant’s workplace address (converted to latitude and longitude coordinates) reported at enrollment. The average daily O_3_ concentrations for 3 years prior to each participant’s enrollment date (i.e., the date of the first medical examination at enrollment) were calculated as a measure of long-term O_3_ exposure levels in this study. We also considered 2-year and 1-year average daily O_3_ concentrations in the sensitivity analysis. In addition, the individual exposure levels of PM_2.5_ and NO_2_ were assessed to investigate the potential confounding effects of other air pollutants. A detailed description of the exposure assessment can be found in a previous study [25].

### Outcome assessment and definition

The primary outcome of this study was incident hypertension, which is defined as SBP ≥ 140 mmHg and/or DBP ≥ 90 mmHg [27, 28] or self-reporting doctor-diagnosed hypertension at the follow-up. Moreover, to analyze the detailed effects of O_3_ exposure on blood pressure, we considered changes in DBP, SBP and two derived components of blood pressure measurements, i.e., PP and MAP, at the first (baseline) versus the last medical examination (last minus first) as secondary outcomes.

SBP and DBP were measured at the baseline and subsequent follow-up medical examinations by medical professionals with the participants in a sitting position for the right arm after 5 min of rest, using a blood pressure monitor (Kenz-AC OSC, Japan). Two readings were taken, 30 s apart, and a third measurement was conducted if the first two reads differed by more than 10 mmHg. The average of the two closest readings was recorded. PP was calculated as the difference between SBP and DBP values, i.e., $PP = SBP - DBP.$ MAP was defined as the average pressure in a patient’s arteries during one cardiac cycle and estimated using SBP and DBP with the following formula [29]:

$${\text{MAP}} = (2 \times {\text{DBP}} + {\text{SBP}})/3\,{\text{or}}\,{\text{MAP}} = {\text{DBP}} + ({\text{SBP}} - {\text{DBP}})/3.$$

### Covariates

Potential confounders and effect modifiers including the participants’ sociodemographic characteristics (age, sex, marital status, and education level), personal history of hypertension, diabetes, cardiovascular diseases, dyslipidemia, cancer, lifestyle factors (smoking, alcohol drinking, habitual night sleep duration and physical exercise), personal measures against air pollution in smog days (mask and air purifier usage) and indoor air pollution (cumulative daily cooking time) were collected using a face-to-face questionnaire at the baseline survey. Age was calculated by subtracting the date of birth from the date of the baseline medical examination. Coronary heart disease (CHD) and cancer were self-reported, and the criteria for the diagnosis of diabetes was self-reported or fasting blood glucose (FBG) ≥ 7.0 mmol/L [30], while dyslipidemia was diagnosed according to the Chinese guideline for the management of dyslipidemia in adults [31], which defined dyslipidemia as triglyceride (TG) ≥ 2.3 mmol/L and/or total cholesterol (TC) ≥ 6.2 mmol/L and/or low-density lipoprotein cholesterol (LDL-C) ≥ 4.1 mmol/L and/or high-density lipoprotein cholesterol (HDL-C) < 1.0 mmol/L, or self-reported doctor-diagnosed dyslipidemia. Sleep duration was assessed using the Pittsburgh Sleep Quality Index and categorized as short (< 7 h per night), optimal (7–8 h per night), and long sleep duration (> 8 h per night). Daily cooking time was divided into three categories: never (0 h), occasional (0–1 h) and frequent cooking (> 1 h). With regard to the personal measures against air pollution, we recorded the use of masks and air purifiers as regular use or not. Smoking status was categorized as never, current and former smoker. Smoking was defined as having smoked continuously at least 1 cigarette per day for more than 6 months, while those who had quit smoking for more than 6 months were considered to be former smokers. Alcohol drinking was defined as consuming alcohol at least once a week. Those who had quit drinking alcohol for a sustained period of a half year or longer by the time of the interview were considered former alcohol drinkers. Physical exercise was defined as exercising more than 3 times per week and for more than 30 min per session. Height (to the nearest 0.1 cm) and weight (to the nearest 0.1 kg) were measured with the participant in light clothing and without shoes using a calibrated stadiometer (GL-310, Seoul, Korea). BMI was calculated as weight (kg) divided by the square of height (m^2^). Due to a small amount of missing data, missing covariates were imputed using single imputation.

### Statistical analysis

For each included study participant, the follow-up duration was defined as the period between study entry (date of the first medical examination) and the endpoint, e.g., the occurrence of a hypertension event, loss to follow-up, or the end of the study, whichever occurred first. Person-years were calculated as the total sum of the number of years that each study participant was followed from enrollment to the endpoint. Descriptive statistics were calculated to assess participants’ clinical characteristics and covariates at baseline. Continuous variables were expressed as mean with corresponding standard deviation (SD), and categorical variables were expressed as frequencies and percentages.

To examine the effects of air pollutant exposure on hypertension, we fitted mixed-effects Cox proportional hazards models with random intercepts for each workplace nested within the city, i.e., nested frailty models [32–34]. The participants were divided into four groups based on the quartiles of O_3_ exposure concentrations (Table 2), which were denoted as Q1 (the first quartile group), Q2 (the second quartile group), Q3 (the third quartile group) and Q4 (the fourth quartile group), respectively. Model parameters were estimated with penalized partial likelihood method. Starting with the model (Model 1) with only the O_3_ concentrations quartile groups as explanatory variables (Q1 was set as the reference), we then evaluated the effects of O_3_ exposure after adjusting for different sets of covariates. Based on Model 1, the sociodemographic characteristics (age, sex, marital status, and education level) were included in Model 2. BMI was further included in Model 3. Model 4 additionally adjusted for the family history of hypertension. Model 5 further adjusted for indoor air pollution and lifestyle factors, including daily cooking time, sleep duration, smoking, alcohol drinking, and participation in physical exercise. Personal protective measures against air pollution (i.e., use of masks and air purifiers during air pollution) were additionally included in Model 6. Finally, Model 7 (the full model) was constructed incorporating biochemical markers (FBG, TG, TC, LDL-C, and HDL-C) and chronic diseases (diabetes, CHD, dyslipidemia and cancer).

For the four continuous secondary outcomes, i.e., the changes in DBP, SBP, PP, and MAP, linear mixed-effects models with nested random intercepts (Model 1 to Model 7 by sequentially adding different sets of covariates as described above) were used.

Stratified analyses were conducted by sex (male and female), age (≤ 44 and > 44 years) and BMI (< 25 and ≥ 25 kg/m^2^), respectively. Sensitivity analyses were further performed to assess the robustness of the associations found between O_3_ exposure and five outcomes in the setting of Model 7. First, we excluded the self-reported physician-diagnosed incident cases of hypertension during follow-up. Second, we took into account the potential confounding effects of other air pollutants such as PM_2.5_ and NO_2_, which have been reported to correlate with O_3_ [35, 36] and also affect blood pressure [37, 38]. Two-pollutant and three-pollutant models were then constructed by introducing one of the two pollutants from PM_2.5_ and NO_2_. Third, the biochemical indicators (FBG, TG, TC, LDL-C, and HDL-C) were substituted with binary variables that represented diabetes and dyslipidemia. Fourth, generalized additive mixed models (GAMMs) were used to investigate the unknown but possible nonlinear concentration-response relationships between O_3_ exposure and five blood pressure-related outcomes. The parameters of the GAMMs were estimated using the restricted maximum likelihood method (REML) [39], and the penalized cubic splines were used to fit the smooth curves, with the effective degrees of freedom automatically estimated by the Akaike information criterion (AIC). Finally, considering that some participants may have worked at the reported organization/institution/company for less than three years, the average daily O_3_ concentrations for one and two years prior to their first medical examination were also calculated as measures of O_3_ exposure levels to be used in the GAMMs.

Statistical analyses were performed using R software (version: 4.2.1) with the packages of “coxme” for fitting nested frailty models, “lmerTest” for fitting multilevel linear mixed-effects models, and “mgcv” for generalized additive model analysis. Statistical tests were two-sided with *P* values < 0.05 considered statistically significant.

## Results

### Descriptive characteristics of the cohort

**Fig. 2:**
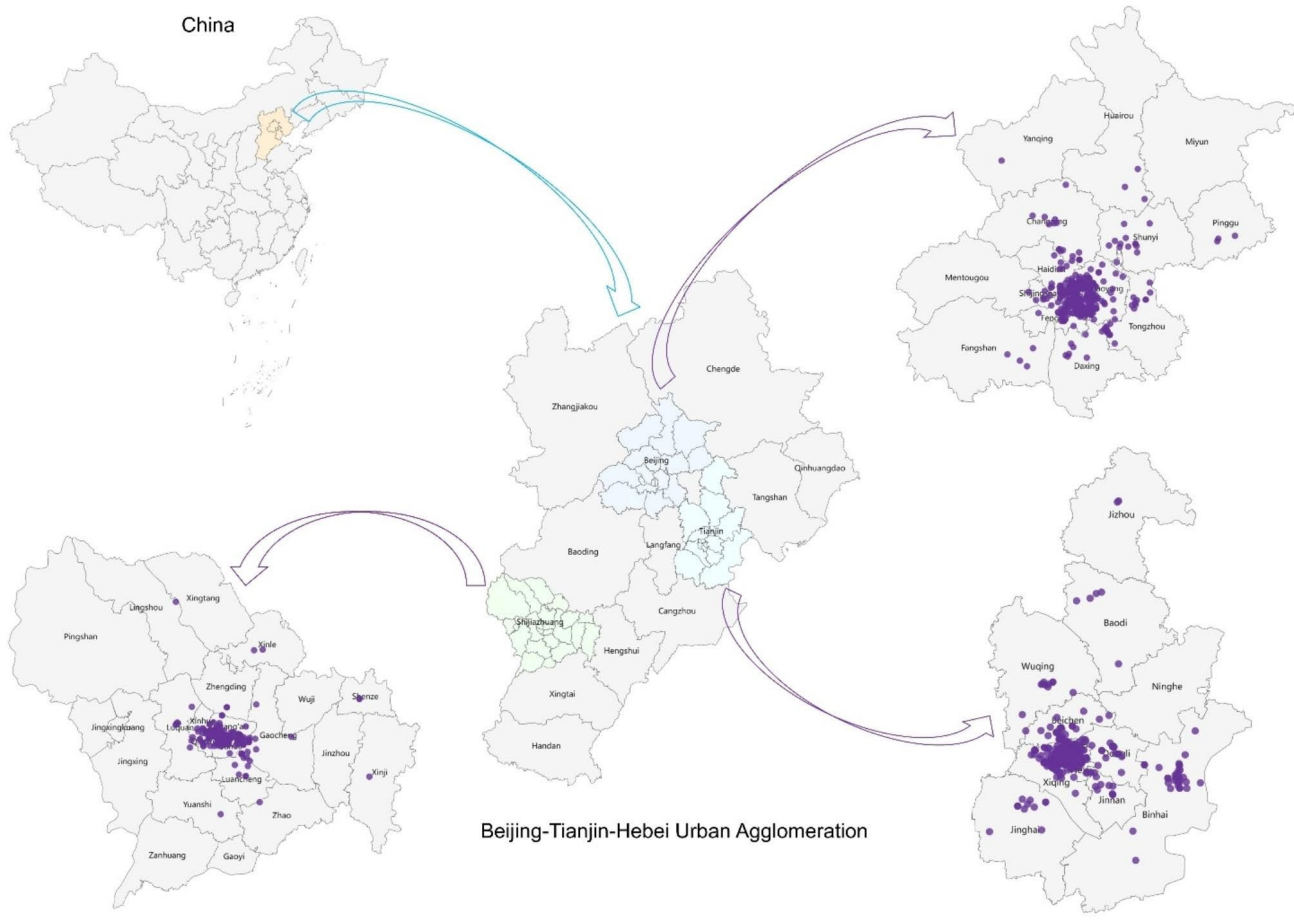
Geographical distribution of study participants’ workplaces in the Beijing-Tianjin-Hebei urban agglomeration

A total of 16,630 participants free of hypertension at baseline from 1,176 organizations, institutions and companies (Fig. 2) were included in the current study. The demographic characteristics of study participants at baseline were summarized in Table 1. All participants were aged between 18 and 65 years, with an average (SD) of 38.87 (9.63) years, and 53.76% were female. The mean (SD) values of participants’ DBP, SBP, PP, and MAP at baseline were 72.49 (8.74), 115.06 (11.74), 42.56 (9.15), and 86.68 (8.85) mmHg, respectively; the mean (SD) concentration of participants’ long-term O_3_ exposure was 45.26 (2.70) ppb (Table 2).

**Table 1:** Baseline characteristics of the participants

Characteristics or variables	Mean (SD) or *n* (%)^a^
Sociodemographic characteristics	
Age (years)	38.87 (9.63)
Sex	
Female	8,940 (53.76%)
Male	7,690 (46.24%)
Marital status	
Single	2,754 (16.56%)
In a current marriage	13,682 (82.27%)
Divorced or widowed	194 (1.17%)
Education level	
High school or below	2,569 (15.45%)
College or undergraduate	10,270 (61.76%)
Postgraduate	3,791 (22.80%)
Family history of hypertension	
Negative	9,841 (59.18%)
Positive	5,815 (34.97%)
Unknown	974 (5.86%)
Indoor air pollution	
Daily cooking time (hours)	
0	5,211 (32.02%)
0–1	7,570 (46.52%)
>1	3,492 (21.46%)
Lifestyle factors	
Night sleep duration (hours/day)	
<7	1,058 (7.05%)
7–8	11,785 (78.52%)
>8	2,165 (14.43%)
Smoking	
Never	13,479 (81.05%)
Current	2,758 (16.58%)
Former	393 (2.36%)
Alcohol drinking	
Never	12,847 (77.25%)
Current	3,619 (21.76%)
Former	164 (0.99%)
Physical exercise	
No	11,701 (70.36%)
Yes	4,929 (29.64%)
Personal measures against air pollution	
Mask usage	
No	11,553 (71.01%)
Yes	4,716 (28.99%)
Air purifier usage	
No	11,012 (67.67%)
Yes	5,262 (32.33%)
Clinical characteristics	
DBP (mmHg)	72.49 (8.74)
SBP (mmHg)	115.06 (11.74)
PP (mmHg)	42.56 (9.15)
MAP (mmHg)	86.68 (8.85)
BMI (kg/m^2^)	23.69 (3.45)
Biochemical indicators	
FBG (mmol/L)	23.69 (3.45)
TG (mmol/L)	5.12 (0.99)
TC (mmol/L)	1.31 (1.04)
LDL-C (mmol/L)	4.67 (0.87)
HDL-C (mmol/L)	2.89 (0.72)
Chronic diseases	
Diabetes	
No	15,928 (96.63%)
Yes	555 (3.37%)
Dyslipidemia	
No	11,836 (74.01%)
Yes	4,157 (25.99%)
CHD	
No	16,548 (99.51%)
Yes	82 (0.49%)
Cancer	
No	16,561 (99.59%)
Yes	69 (0.41%)

Note: DBP, diastolic blood pressure; SBP, systolic blood pressure; PP, pulse pressure; MAP, mean arterial pressure; BMI, body mass index; FBG, fasting blood glucose; TG, triglyceride; TC, total cholesterol; LDL-C, low-density lipoprotein cholesterol; HDL-C, high-density lipoprotein cholesterol; CHD, coronary heart disease; SD, standard deviation

^a^ The variables under investigation, including BMI, daily cooking time, night sleep duration, mask usage, air purifier usage, FBG, TG, TC, LDL-C, HDL-C, diabetes, and dyslipidemia, had varying amounts of missing data. Specifically, 165 (0.99%), 357 (2.15%), 1,622 (9.75%), 361 (2.17%), 356 (2.14%), 149 (0.90%), 646 (3.88%), 640 (3.85%), 813 (4.89%), 813 (4.89%), 147 (0.88%) and 637 (3.83%) missing values were observed, respectively. Percentages may not add to 100% due to rounding

**Table 2:** Distribution of the participants’ 3-year average daily air pollutant exposure concentrations

Pollutant	Mean	SD	Minimum	25th percentile	Median	75th percentile	Maximum
O_3_ (ppb)	45.26	2.70	35.43	44.09	46.19	47.07	50.96
PM_2.5_ (µg/m^3^)	70.10	7.53	47.02	64.88	67.38	76.97	93.04
NO_2_ (ppb)	25.81	1.00	21.04	25.08	25.90	26.48	28.13

Note: SD, standard deviation; O_3_, ozone; PM_2.5_, particulate matter with aerodynamic diameter ≤ 2.5 μm; NO_2_, nitrogen dioxide; ppb, parts per billion

During the follow-up of 21,946 person-years, we identified 1,635 (9.83%) incident hypertension cases. The cumulative incidence of hypertension was 7.11 (95% CI: 6.76, 7.47) per 100 person-years (Fig. S1), with 10.53 (95% CI: 9.96, 11.11) per 100 person-years for men and 4.32 (95% CI: 3.97, 4.67) per 100 person-years for women (*P* < 0.001). The cumulative incidence of hypertension was higher in individuals aged > 44 years (10.98 vs. 5.41 per 100 person-years, *P* < 0.001) or with a BMI ≥ 25 (11.47 vs. 5.10 per 100 person-years, *P* < 0.001).

### Associations between long-term O_3_ exposure and hypertension

**Fig. 3:**
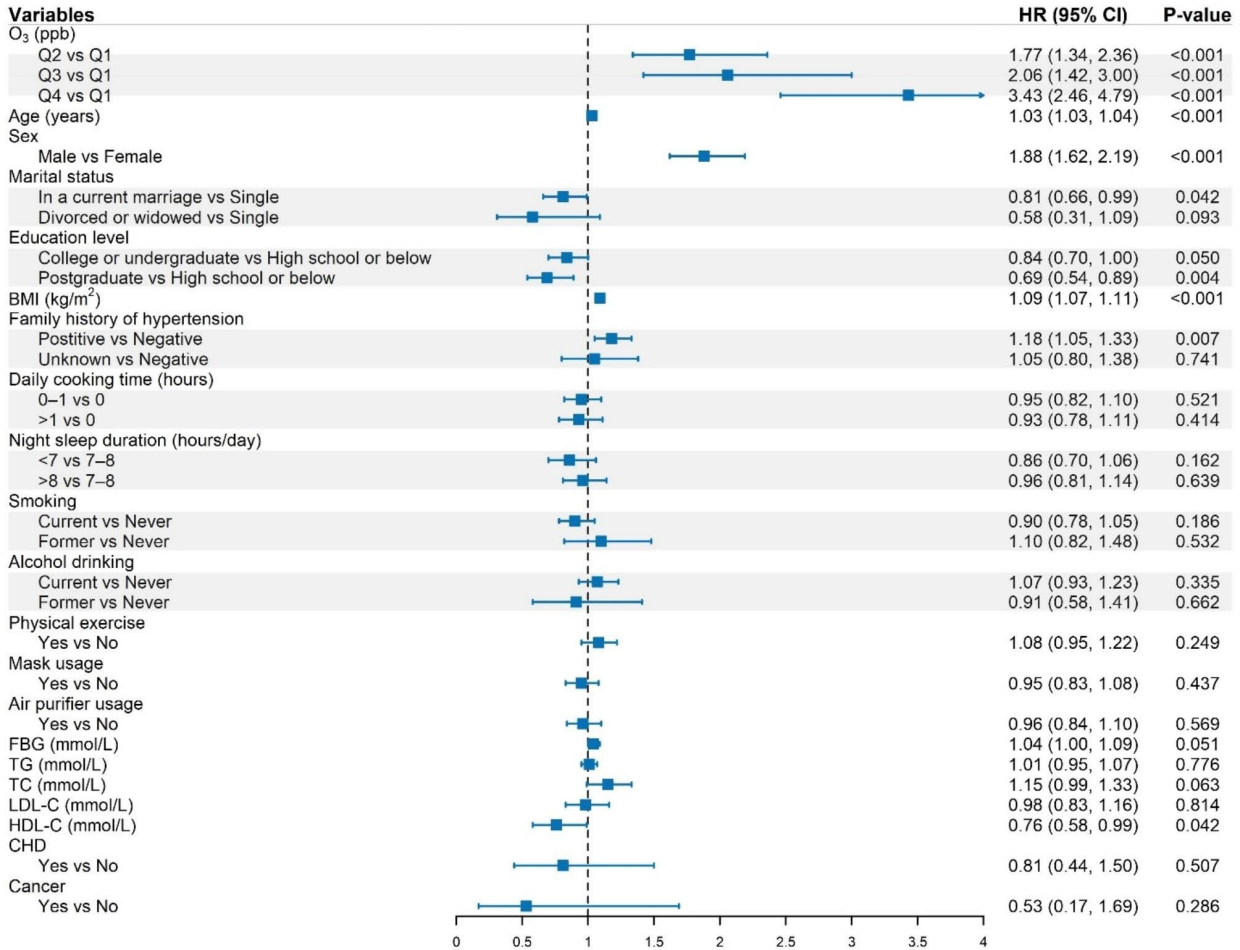
Estimated hazard ratios with 95% CIs of long-term O_3_ exposure and the covariates derived from the full nested frailty model. The arrow denotes that the 95% CIs of the estimated effect exceeds the display range of the graph, and the excess part is indicated by an arrow. Note: HR, hazard ratio; CI, confidence interval; O_3_, ozone; Q1–Q4, the first to the fourth quartile groups of O_3_ exposure concentrations; ppb, parts per billion; BMI, body mass index; FBG, fasting blood glucose; TG, triglyceride; TC, total cholesterol; LDL-C, low-density lipoprotein cholesterol; HDL-C, high-density lipoprotein cholesterol; CHD, coronary heart disease

The results of the nested mixed-effects model analysis in Table 3 suggested significant impacts of long-term exposure to O_3_ on incident hypertension. Compared with the first quartile group (Q1) of the O_3_ concentration, the estimated hazard ratios (HRs) and 95% confidence intervals (CIs) were 2.16 (95% CI: 1.68, 2.79), 2.22 (95% CI: 1.57, 3.15), and 3.85 (95% CI: 2.86, 5.18) (all *P* < 0.001) for the Q2, Q3 and Q4 of the O_3_ concentrations in the crude model where only O_3_ concentrations were included as explanatory variable (Model 1). The significant associations changed slightly and persisted in the multivariable models with further adjustment for sociodemographic characteristics (Model 2), BMI (Model 3), family history of hypertension (Model 4), indoor air pollution and lifestyle factors (Model 5), personal measures against air pollution in smog days (Model 6), and serum lipids, FBG, and chronic diseases (Model 7) (all *P* < 0.001). In the fully adjusted model (Model 7), the HRs were attenuated slightly to 1.77 (95% CI: 1.34, 2.36), 2.06 (95% CI: 1.42, 3.00), and 3.43 (95% CI: 2.46, 4.79) for Q2 to Q4 of O_3_ exposure, respectively. Detailed results of the full model are shown in Fig. 3.

**Table 3:** Relationship between long-term O_3_ exposure concentrations and hypertension, DBP, SBP, PP and MAP derived from nested mixed-effects models

Model		Hypertension	DBP	SBP	PP	MAP
		HR (95% CI)	${\beta ^{\text{a}}}$(95% CI)	${\beta ^{\text{a}}}$(95% CI)	${\beta ^{\text{a}}}$(95% CI)	${\beta ^{\text{a}}}$(95% CI)
Model 1^b^	Q1	Ref	Ref	Ref	Ref	Ref
	Q2	2.16 (1.68, 2.79) *	1.06 (0.50, 1.62) *	3.77 (2.99, 4.55) *	2.63 (1.97, 3.30) *	1.95 (1.40, 2.50) *
	Q3	2.22 (1.57, 3.15) *	0.59 (− 0.16, 1.34)	3.11 (2.07, 4.15) *	2.35 (1.56, 3.15) *	1.46 (0.72, 2.19) *
	Q4	3.85 (2.86, 5.18) *	0.71 (0.09, 1.34) *	3.30 (2.43, 4.17) *	2.48 (1.79, 3.16) *	1.60 (0.98, 2.22) *
Model 2^c^	Q1	Ref	Ref	Ref	Ref	Ref
	Q2	2.11 (1.64, 2.72) *	1.02 (0.46, 1.58) *	3.72 (2.94, 4.50) *	2.61 (1.95, 3.28) *	1.90 (1.35, 2.45) *
	Q3	2.36 (1.66, 3.35) *	0.59 (− 0.16, 1.34)	3.13 (2.09, 4.16) *	2.35 (1.55, 3.14) *	1.45 (0.72, 2.18) *
	Q4	3.96 (2.93, 5.34) *	0.70 (0.07, 1.33) *	3.34 (2.47, 4.21) *	2.51 (1.82, 3.19) *	1.60 (0.98, 2.22) *
Model 3^d^	Q1	Ref	Ref	Ref	Ref	Ref
	Q2	2.01 (1.56, 2.60) *	1.03 (0.47, 1.59) *	3.60 (2.82, 4.38) *	2.50 (1.84, 3.17) *	1.87 (1.31, 2.42) *
	Q3	2.31 (1.63, 3.28) *	0.55 (− 0.20, 1.31)	2.99 (1.95, 4.02) *	2.29 (1.49, 3.08) *	1.38 (0.64, 2.11) *
	Q4	3.76 (2.78, 5.09) *	0.67 (0.03, 1.30) *	3.23 (2.36, 4.10) *	2.46 (1.77, 3.15) *	1.54 (0.92, 2.16) *
Model 4^e^	Q1	Ref	Ref	Ref	Ref	Ref
	Q2	2.00 (1.55, 2.58) *	1.02 (0.46, 1.59) *	3.59 (2.81, 4.37) *	2.50 (1.83, 3.16) *	1.86 (1.30, 2.41) *
	Q3	2.30 (1.62, 3.27) *	0.56 (− 0.20, 1.31)	2.99 (1.96, 4.03) *	2.31 (1.51, 3.10) *	1.38 (0.64, 2.12) *
	Q4	3.76 (2.78, 5.09) *	0.67 (0.04, 1.31) *	3.24 (2.37, 4.11) *	2.48 (1.79, 3.16) *	1.55 (0.92, 2.17) *
Model 5^f^	Q1	Ref	Ref	Ref	Ref	Ref
	Q2	1.85 (1.40, 2.43) *	0.99 (0.39, 1.59) *	3.53 (2.70, 4.36) *	2.44 (1.74, 3.15) *	1.81 (1.23, 2.40) *
	Q3	2.21 (1.52, 3.19) *	0.54 (− 0.26, 1.34)	3.06 (1.97, 4.15) *	2.29 (1.46, 3.11) *	1.39 (0.61, 2.17) *
	Q4	3.65 (2.64, 5.06) *	0.56 (− 0.12, 1.24)	3.20 (2.27, 4.13) *	2.47 (1.74, 3.20) *	1.46 (0.79, 2.12) *
Model 6^ g^	Q1	Ref	Ref	Ref	Ref	Ref
	Q2	1.85 (1.40, 2.43) *	0.98 (0.38, 1.59) *	3.53 (2.70, 4.36) *	2.47 (1.76, 3.17) *	1.81 (1.22, 2.40) *
	Q3	2.20 (1.52, 3.18) *	0.51 (− 0.29, 1.31)	3.05 (1.95, 4.14) *	2.31 (1.48, 3.14) *	1.36 (0.58, 2.14) *
	Q4	3.61 (2.61, 5.00) *	0.53 (− 0.15, 1.21)	3.17 (2.24, 4.11) *	2.48 (1.74, 3.21) *	1.43 (0.76, 2.09) *
Model 7^ h^	Q1	Ref	Ref	Ref	Ref	Ref
	Q2	1.77 (1.34, 2.36) *	0.65 (0.01, 1.30) *	2.88 (2.00, 3.77) *	2.12 (1.36, 2.87) *	1.39 (0.76, 2.02) *
	Q3	2.06 (1.42, 3.00) *	0.28 (− 0.54, 1.10)	2.49 (1.36, 3.61) *	2.03 (1.18, 2.87) *	1.04 (0.24, 1.84) *
	Q4	3.43 (2.46, 4.79) *	0.33 (− 0.38, 1.03)	2.61 (1.64, 3.58) *	2.14 (1.38, 2.90) *	1.12 (0.43, 1.82) *

Note: DBP, diastolic blood pressure; SBP, systolic blood pressure; PP, pulse pressure; MAP, mean arterial pressure; HR, hazard ratio; CI, confidence interval; Q1–Q4, the first to the fourth quartile groups of O_3_ exposure concentrations

^a^$\beta$ represents the average increase in the outcomes compared to Q1

^b^ Model 1 considered only the quartile groups of O_3_ exposure concentrations as explanatory variable

^c^ Model 2 adjusted for age, sex, marital status and education level

^d^ Model 3 adjusted for variables in Model 2 plus BMI

^e^ Model 4 adjusted for variables in Model 3 plus family history of hypertension

^f^ Model 5 adjusted for variables in Model 4 plus daily cooking time, night sleep duration, smoking, alcohol drinking and physical exercise

^g^ Model 6 adjusted for variables in Model 5 plus mask usage and air purifier usage

^h^ Model 7 adjusted for variables in Model 6 plus FBG, TG, TC, LDL-C, HDL-C, CHD and cancer

* P-value < 0.05

The results for the associations between O_3_ exposure and four secondary continuous outcomes were also presented in Table 3. When DBP was used as the outcome, the full model (Model 7) yielded an estimated coefficient (the incremental effect relative to Q1) of 0.65 (95% CI: 0.01, 1.30) for Q2, 0.28 (95% CI: −0.54, 1.10) for Q3 and 0.33 (95% CI: −0.38, 1.03) for Q4, with only the coefficient of Q2 being statistically significant; whereas, when SBP was used as the outcome, the estimated coefficients for Q2–Q4 were 2.88 (95% CI: 2.00, 3.77), 2.49 (95% CI: 1.36, 3.61), and 2.61 (95% CI: 1.64, 3.58), respectively; when PP was used as the outcome, the estimated coefficients were 2.12 (95% CI: 1.36, 2.87), 2.03 (95% CI: 1.18, 2.87), and 2.14 (95% CI: 1.38, 2.90), respectively; when MAP was used as the outcome, the estimated coefficients were 1.39 (95% CI: 0.76, 2.02), 1.04 (95% CI: 0.24, 1.84) and 1.12 (95% CI: 0.43, 1.82), respectively. The coefficients of the Q2 to Q4 for SBP, PP, and MAP were all statistically significant.

Detailed parameter estimation results for Models 1 to 7 based on the five outcomes were presented in Tables S1 to S5.

### Results of stratified analysis

**Fig. 4:**
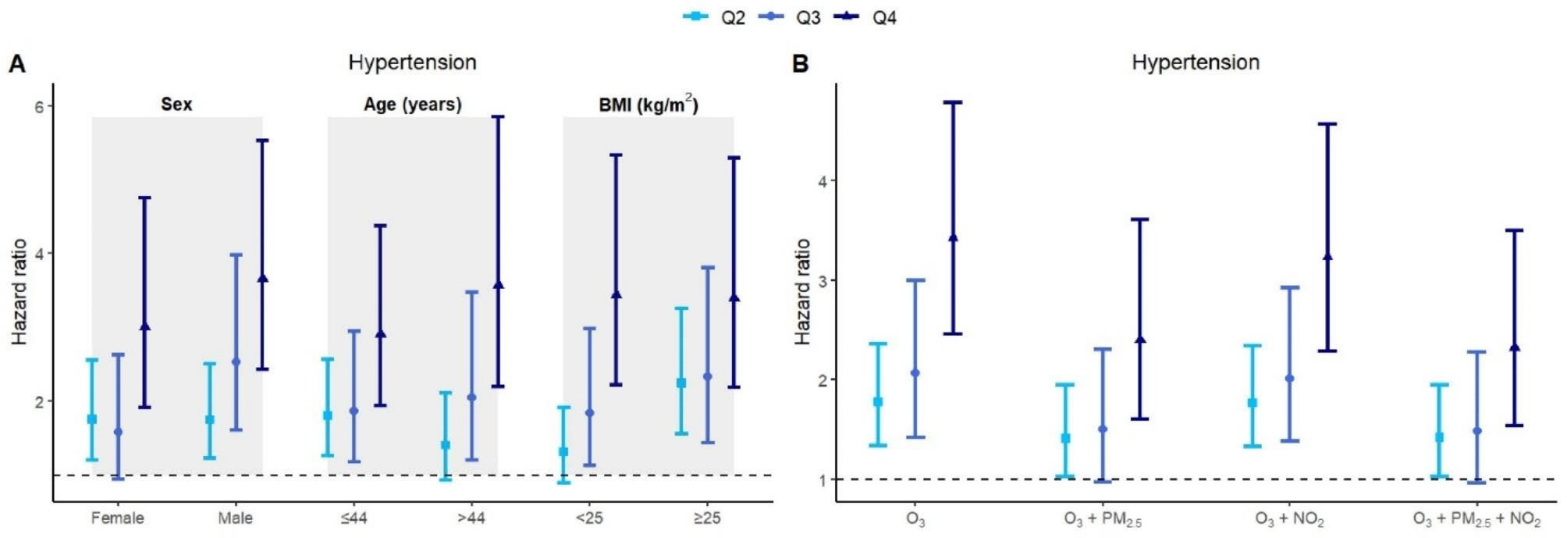
Estimated HRs with 95% CIs of long-term O_3_ exposure for hypertension in different subgroups derived from fully nested frailty models and single-, two- and three-pollutant models. The panel **A** shows stratified analysis according to sex (male and female), age (≤ 44 and > 44 years) and BMI (< 25 and ≥ 25 kg/m^2^) for the outcome of hypertension. The panel **B** shows two-pollutant and three-pollutant models involving the pollutants of PM_2.5_ and NO_2_. Note: O_3_, ozone; PM_2.5_, particulate matter with aerodynamic diameter ≤ 2.5 μm; NO_2_, nitrogen dioxide; BMI, body mass index; Q2–Q4, the second to the fourth quartile groups of O_3_ exposure concentrations

Based on the setting of Model 7, we first performed stratified analysis by sex (male and female), age (≤ 44 and > 44 years) and BMI (< 25 and ≥ 25 kg/m^2^). Panel A of Fig. 4 showed that the HRs for Q2–Q4 were significant in all subgroups, except that the HR for Q2 in the older age group and lower BMI group, and Q3 in females were marginally significant. The risk of exposure to O_3_ in the Q3 and Q4 quartile groups was higher in males than in females (with overlapping 95% CIs). In the older population, Q4 had a greater effect on hypertension than in the younger population (with overlapping 95% CIs), whereas Q2 and Q3 tended to have greater effects in the overweight population (with overlapping 95% CIs). We also considered the interaction effects of quantiles of O_3_ exposure and population subgroups (sex, age and BMI). As shown in Table S6, the interactions between Q2 and sex (*P* = 0.047), Q2 and Q3, and BMI (*P* value was 0.026 and 0.025, respectively) were significant. The results of stratified analysis for four secondary outcomes (DBP, SBP, PP, and MAP) were presented in Fig. S2.

### Results of sensitivity analysis

To minimize potential effects on blood pressure indicators by medications, the full nested mixed-effects models were re-fitted after excluding the 98 subjects who reported doctor-diagnosed hypertension during the follow-up. The results, which were presented in Table S7, showed that the estimated effects of exposure to O_3_ concentrations quartiles for five outcomes were almost unchanged.

We constructed two-pollutant and three-pollutant models by introducing one or both of the air pollutants PM_2.5_ and NO_2_ into the full model. The estimated hazard ratios and 95% CIs for the O_3_ exposure quartiles differed across models, but the overall trends were similar, as shown in panel B of Fig. 4. The results of the two-pollutant and three-pollutant models for four secondary outcomes were shown in Fig. S3.

Moreover, we replaced biomarker indicators (i.e., FBG, TG, TC, LDL-C, and HDL-C) with diabetes and dyslipidemia and re-estimated Model 7. The results (Table S8) showed that the effect of O_3_ exposure on the five outcomes remained unchanged and comparably robust.

### Concentration-response associations

**Fig. 5:**
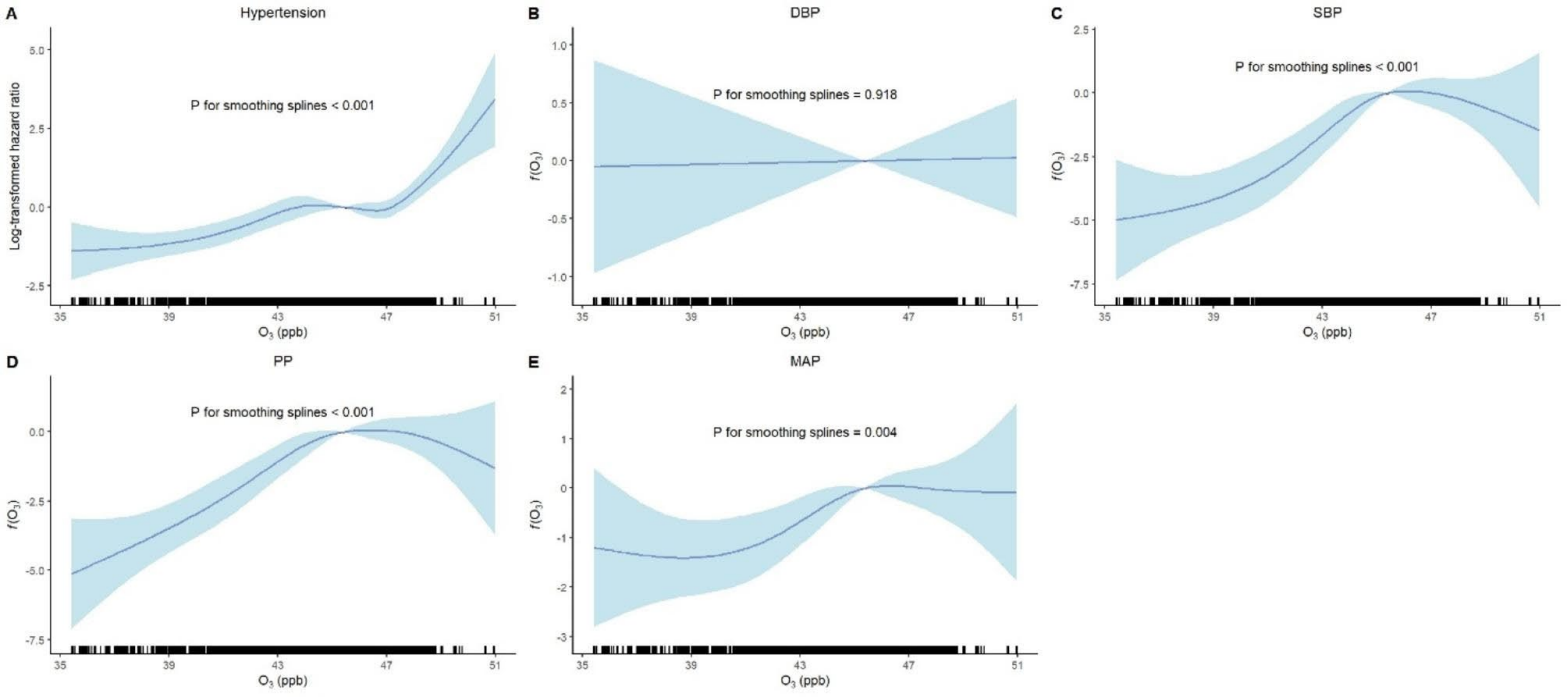
Concentration-response curves of the association between concentrations of long-term O_3_ exposure and hypertension, DBP, SBP, PP and MAP. Analyses are adjusted for age, sex, marital status, education level, BMI, family history of hypertension, daily cooking time, night sleep duration, smoking, alcohol drinking, mask usage, air purifier usage, FBG, TG, TC, LDL-C, HDL-C, CHD and cancer. Effect estimates are indicated by solid lines and 95% confidence intervals by shaded areas. Darker colors in the lower bars represent a higher sample clustering. Hazard ratios are on a logarithmic scale. Note: DBP, diastolic blood pressure; SBP, systolic blood pressure; PP, pulse pressure; MAP, mean arterial pressure; O_3_, ozone; ppb, parts per billion

We also used the GAMMs to flexibly model and visualize the possible non-linear relationship between O_3_ exposure concentrations and five outcomes (Fig. 5). The relationship between O_3_ exposure concentrations and hypertension varied in three stages, i.e., slowly increasing (to the first quartile, 44.09 ppb), almost flat (to the third quartile, 47.07 ppb), and rapidly increasing. There was no clear increasing or decreasing trend for DBP, and the test for non-linearity was not significant (*P* = 0.918). The relationship between O_3_ exposure concentrations and the other three secondary outcomes showed an overall increasing trend (increased first and then became flat). The patterns of the estimated concentration-response curves were consistent with the findings in Table 3.

Finally, the GAMMs were analyzed by using the average daily O_3_ concentrations for one and two years (instead of three years) prior to their first medical examination as a measure of O_3_ exposure. The results are shown in Fig. S4 to S5.

## Discussion

To the best of our knowledge, the current prospective cohort study is the first to report positive and non-linear associations of long-term exposure to O_3_ with incident hypertension, elevated SBP, DBP, PP and MAP among the general employed population in a highly polluted area. Compared with the Q1 of O_3_ concentrations, the HRs of hypertension for the Q2 to Q4 were 1.77 (95% CI: 1.34, 2.36), 2.06 (95% CI: 1.42, 3.00), and 3.43 (95% CI: 2.46, 4.79), respectively. SBP, PP and MAP significantly increased by 2.49–2.88, 2.03–2.14, and 1.04–1.39 mmHg with O_3_ concentrations of Q2–Q4 compared to O_3_ concentrations at Q1, while DBP increased modestly by 0.65 mmHg only at O_3_ concentrations of Q2. The robustness of our findings was confirmed by the consistent results when one or both of two air pollutants (PM_2.5_ and NO_2_) were added to the models and those who reported doctor-diagnosed incident hypertension during the follow-up were excluded. Stratification analyses indicated that the long-term impacts of O_3_ exposure persisted regardless of sex, age and BMI, while males, overweight and obese individuals were more vulnerable. These findings might improve the current understanding of the role of O_3_ exposure in the occurrence of hypertension and blood pressure modulation, and further promote the formation of targeted public health policies to improve public cardiovascular health.

Although the relationship between ambient pollutants and blood pressure or hypertension has been enormously investigated, the impacts of long-term exposure to O_3_ on hypertension were much less addressed [4, 40, 41]. Prior to our study, several cross-sectional studies reported inconsistent relationships between long-term exposure to O_3_ with prevalent hypertension, however, its association with incident hypertension was seldom investigated [11, 40]. One prospective cohort study in black women found that every 6.7 ppb increment of O_3_ exposure was associated with a 9% (HR: 1.09; 95% CI: 1.00, 1.18) higher risk of incident hypertension based on the single-pollutant model with adjustment for potential covariates. However, the estimated HR attenuated to non-significance (HR: 1.04; 95% CI: 0.94, 1.15) once another pollutant (NO_2_ and PM_2.5_) was further added to the model, leaving the relationship still unsolved [16]. Another longitudinal study among African American with a high (56%) prevalence of hypertension failed to observe any significant association of 1-year O_3_ concentrations (RR: 0.91; 95% CI: 0.77, 1.08) or 3-year O_3_ concentrations (RR: 0.93; 95% CI: 0.84, 1.02) with incident hypertension [14]. It is important to note that these studies assessed the O_3_ exposure levels of the study subjects’ residential addresses. Given the diurnal pattern of O_3_ concentrations, i.e., higher concentrations during the day and lower concentrations at night [18, 19], the studies above may have misestimated the O_3_ levels that caused adverse health effects on the study subjects. Moreover, the low levels of O_3_ exposure and small study sample sizes may have reduced the power of the aforementioned longitudinal studies, failing to detect any positive associations between O_3_ exposure and the development of hypertension. Our study, which restricted the participants to working adults, with a large sample size, prospective design and high levels of O_3_ exposure, found a positive association between 3-year exposure to O_3_ at workplace with incident hypertension. The results remained consistent and statistically significant when we applied two-pollutant models by adding PM_2.5_ and NO_2_. Our findings not only confirmed the overall positive association between O_3_ exposure and the risk of incident hypertension but also indicated that this association was robust against other air pollutants.

Although blood pressure levels are a better measure of the health risks associated with blood pressure than hypertensive status, only a few studies have evaluated the effect of long-term O_3_ exposure on blood pressure levels and the results were inclusive [15, 40]. Moreover, the longitudinal effects of long-term O_3_ exposure on blood pressure indicators have been rarely documented [40]. The aforementioned prospective study in highly hypertensive African Americans, due to the smaller variation in O_3_ exposure (IQR = 0.7 ppb) and sample size (*n* = 4,105), detected only marginal and non-clinically relevant effect of 3-year O_3_ concentrations on blood pressure indicators, e.g., SBP, DBP, MAP increased by 0.20 (95% CI: 0.001, 0.39), 0.14 (95% CI: 0.03, 0.25) and 0.16 (95% CI: 0.04, 0.29) mmHg for every interquartile increment in O_3_ concentrations, while the increment of PP was non-significant (0.05 (95% CI: −0.11, 0.20) mmHg). The impact of long-term O_3_ exposure on blood pressure remains to be clarified. Nevertheless, the current study, with a much larger sample size and greater variation in O_3_ exposure, detected considerable significant increases in SBP, DBP, MAP and PP, which may not only contribute to a more profound understanding of the effects of O_3_ exposure on blood pressure indicators, but may also have potential clinical relevance.

Notably, we observed the most substantial increases in SBP, where the increments were 2.88 (95% CI: 2.00, 3.77), 2.49 (95% CI: 1.36, 3.61), and 2.61 (95% CI: 1.64, 3.58) mmHg higher for the second to fourth quartile, respectively, compared with the first quartile of O_3_ exposure concentrations; whereas the least substantial increases were observed in DBP, suggesting that long-term exposure to O_3_ impacts SBP more than DBP. Higher SBP has been consistently associated with increased CVD risk after adjustment for or stratification by DBP, whereas the results about the association between higher DBP and CVD risk are inconsistent after adjustment for or stratification by SBP [42–45]. High SBP has been a vital contributor to death at the global level [1]. Therefore, our findings suggest that the adverse effects of long-term exposure to O_3_ on blood pressure may contribute to deleterious cardiovascular outcomes or death.

Though a few positive findings regarding the effects of long-term O_3_ exposure on elevated SBP, DBP and MAP, no significant findings have been reported regarding the relationship between long-term O_3_ exposure and elevated PP [11, 14, 15]. Determined by the compliance of arteries and the timing and intensity of arterial wave reflections, PP is usually considered as an indicator of arterial stiffness [46]. The current finding, by reporting for the first time a significant effect of long-term O_3_ exposure on elevated PP, may suggest that arterial stiffness is involved in the blood pressure regulation induced by long-term O_3_ exposure, in contrast to previous studies that reported positive correlations between short-term or long-term O_3_ exposure and SBP, DBP and MAP, and smaller or non-significant correlations with several indices of arterial stiffness (including carotid-femoral pulse wave velocity, anterior pressure wave amplitude and augmentation index) [11, 14, 47–49]. Our study provides evidence of a positive relationship between long-term O_3_ exposure and elevated SBP, MAP, and PP, which may improve the current understanding of the role of long-term O_3_ exposure in the regulation of blood pressure and the development of cardiometabolic diseases.

Even though previous studies have mostly assumed a linear relationship [12–16], the detailed shape of the relationship between O_3_ exposure and the risk of hypertension remains a key question that has not been addressed. With a wide range of O_3_ exposure concentrations in our study (35.43 to 50.96 ppb), using generalized additive mixed models, this study revealed a non-linear relationship between O_3_ exposure levels and incident hypertension, where the risk of hypertension increased slowly from 35.43 to 44.09 ppb, while it was at almost a stable level within 44.09 to 47.07 ppb, and then elevated sharply when the concentrations were greater than 47.07 ppb. Previously, mixed results have been found regarding the relationship between O_3_ exposure and cardiovascular outcomes. For example, ecological study suggested a positive correlation between exposure to ambient O_3_ and mortality of cardiovascular disease among the elderly in the Middle East [50], and several recent large cohort studies in the US and China showed that long-term exposure to O_3_ was positively and monotonically related to cardiovascular mortality [5, 51, 52], whereas null or even negative associations were also reported in cohorts from France, Denmark, and the UK [53–55]. Since hypertension is one of the most important risk factors for various cardiovascular outcomes, the non-linear shape of the relationship between O_3_ exposure and hypertension might indicate the presence of non-linear associations between O_3_ exposure and other cardiovascular outcomes.

The stratified analyses indicated that the observed associations between long-term exposure to O_3_ and hypertension remained consistent across sex, age and BMI categories, while sex and BMI interacted with O_3_ and males, overweight, obese and older individuals were more vulnerable to the adverse effects of O_3_. The robust findings were not fully consistent with previous studies. For example, the positive cross-sectional relationship between long-term O_3_ exposure and hypertension was limited to men and was more pronounced in participants older than 65 years old among Chinese adults [12], while the longitudinal evidence from the US suggested the hypertensive impact of long-term exposure to O_3_ was stronger in women [14]. In addition, hypertensive effects of long-term exposure to O_3_ have been reported to be mediated by BMI [11]. The different study designs, sample sizes, approaches to assessing O_3_ exposure and low levels of O_3_ concentrations may be the potential explanation for the null association detected in less vulnerable populations.

Although the potential mechanisms underlying the association between long-term O_3_ exposure and hypertension are not fully understood, systematic inflammation, oxidative stress reactions and endothelial dysfunction may contribute to the complicated mechanisms through which long-term exposure to O_3_ influences blood pressure [48, 56, 57]. O_3_ is a reactive oxygen species (ROS) that can damage cells and tissues [58]. This damage can lead to inflammation, which can in turn contribute to hypertension. In addition, O_3_ can damage the lining of blood vessels (endothelium), contributing to endothelial dysfunction [59]. This is a condition in which the endothelium is less able to regulate blood flow and blood pressure. Moreover, O_3_ exposure may activate the renin-angiotensin-aldosterone system (RAAS), a system that regulates blood pressure. Activation of the RAAS can lead to increased production of angiotensin II, a hormone that constricts blood vessels and raises blood pressure [60]. Third, research has linked O_3_ exposure to changes in levels of certain hormones, such as cortisol and aldosterone, which play a role in regulating blood pressure [61]. Some people may be more susceptible to the effects of O_3_ exposure on blood pressure than others. This may be due to genetic factors [62].

The prospective nature may be the key strength of the current study. By conducting the population-based cohort study, we quantify the impacts of long-term exposure to O_3_ on incident hypertension and blood pressure among the general employed population in a highly polluted area. Second, we focused our study on the working population and estimated their daytime O_3_ exposure levels, i.e., workplace O_3_ exposure levels, rather than nighttime O_3_ exposure levels, i.e., residential O_3_ exposure levels. Considering the diurnal pattern of O_3_ concentration, with a higher concentration in the daytime and a lower concentration at night [18, 19], O_3_ exposure at daytime is what causes health risks. By using workplace O_3_ exposure, we might have controlled measurement bias and improved the power of the study. Third, we transformed the O_3_ exposure concentrations to a four-level categorical variable based on their quartiles and also utilized the GAMMs to detect a non-linear relationship between O_3_ exposure and hypertension and blood pressure indicators, which is vital for a more detailed understanding of the hypertensive effects of O_3_ exposure and provides new perspectives for future studies on the health effects of ambient pollutants. Fourth, only the urban population was included in this study to ensure the reliability of the results. Given the different sources and components of ambient pollutants in urban and rural areas [63], as well as the differences in factors affecting hypertension and susceptibility to hypertension in urban and rural populations [64], the inclusion of both urban and rural populations in the study may introduce additional confounding. Finally, we included the highly prevalent cardiometabolic risk behaviors in contemporary society, i.e., sleep deprivation and being sedentary, as covariates, and also included personal mask-wearing and air purifier use in the model.

Nevertheless, our findings must be interpreted with caution due to several limitations. We did not collect information on or adjust for some factors affecting blood pressure, such as salt intake and dietary patterns, resulting in a confounding bias in the results. Moreover, as in most previous epidemiological studies, we could not account for the geographic mobility of the population during the follow-up period. Third, although we used a two-stage machine learning model, which is likely reduced misclassification compared to simpler methods, there are still inherent disparities between the estimated O_3_ exposure and the actual O_3_ exposure experienced by individuals as the model cannot account for all individual-level factors that may affect O_3_ exposure, such as time spent indoors and outdoor activities.

The current findings are particularly relevant for policymakers and researchers involved in the control of ambient pollution and public health. Although additional studies are needed to further explore the mechanism underlying the relationship between O_3_ exposure and cardiovascular diseases, this study supports the integration of reduction of ambient O_3_ concentration into public health interventions to improve cardiovascular health, especially among vulnerable populations such as males, overweight and obese individuals.

## Conclusions

Our data from the large-scale, prospective cohort in China, for the first time, provide evidence that long-term exposure to ambient O_3_ at workplace is independently and non-linearly associated with an increased risk of incident hypertension and elevated blood pressure among working adults. The associations were robust regardless of age, sex, or BMI, while males, overweight and obese individuals were more vulnerable to the unfavorable effects of O_3_. The findings help improve the current understanding of the long-term hypertensive effect of O_3_ exposure. Considering the high prevalence of hypertension and its associated adverse health outcomes, together with the global prevalence of air pollution, the current findings are seen as more than just a revelation for the prevention of hypertension and may provide a new perspective for improving global public health security.

### Electronic supplementary material

Below is the link to the electronic supplementary material.

Supplementary Material 1

Supplementary Material 2

Supplementary Material 3

Supplementary Material 4

Supplementary Material 5

Supplementary Material 6

Supplementary Material 7

Supplementary Material 8

Supplementary Material 9

Supplementary Material 10

Supplementary Material 11

Supplementary Material 12

Supplementary Material 13

## Data Availability

The datasets are not publicly available because of the private information but could be accessed from the corresponding author with a reasonable request.

## Abbreviations

BMI: Body mass index
BTH: Beijing-Tianjin-Hebei
BTH-MEC: Beijing-Tianjin-Hebei Medical Examination-based Cohort
CHD: Coronary heart disease
CI(s): Confidence interval(s)
DBP: Diastolic blood pressure
FBG: Fasting blood glucose
GAMMs: Generalized additive mixed models
HDL-C: High-density lipoprotein cholesterol
HR(s): Hazard ratio(s)
LDL-C: Low-density lipoprotein cholesterol
MAP: Mean arterial pressure
NO_2_: Nitrogen dioxide
O_3_: Ozone
PM_2.5_: Particulate matter with aerodynamic diameter ≤ 2.5 μm
PP: Pulse pressure
ppb: Parts per billion
Q1: The first quartile group of O_3_ exposure concentrations
Q2: The second quartile group of O_3_ exposure concentrations
Q3: The third quartile group of O_3_ exposure concentrations
Q4: The fourth quartile group of O_3_ exposure concentrations
RAAS: Renin-angiotensin-aldosterone system
SBP: Systolic blood pressure
SD: Standard deviation
TC: Total cholesterol
TG: Triglyceride
