# Supplementary material for: Long-term exposure to ambient ozone at workplace is positively and non-linearly associated with incident hypertension and blood pressure: longitudinal evidence from the Beijing-Tianjin-Hebei medical examination cohort
Source: BMC Public Health. 2023 Oct 16;23:2011. doi: 10.1186/s12889-023-16932-w (PMC10577958; doi:10.1186/s12889-023-16932-w)
Supplement: Supplementary file 1 — Supplementary Material 1 [file 12889_2023_16932_MOESM1_ESM.docx]

**Table S1** Relationship between long-term O_3_ exposure concentrations and hypertension derived from the nested frailty models

| **Model** | **HR (95% CI)** | **P-value** |
| --- | --- | --- |
| Model 1 |  |  |
| O_3_ (Q2 vs Q1) | 2.16 (1.68, 2.79) * | <0.001 |
| O_3_ (Q3 vs Q1) | 2.22 (1.57, 3.15) * | <0.001 |
| O_3_ (Q4 vs Q1) | 3.85 (2.86, 5.18) * | <0.001 |
| Model 2 (Model 1 + Sociodemographic characteristics) |  |  |
| O_3_ (Q2 vs Q1) | 2.11 (1.64, 2.72) * | <0.001 |
| O_3_ (Q3 vs Q1) | 2.36 (1.66, 3.35) * | <0.001 |
| O_3_ (Q4 vs Q1) | 3.96 (2.93, 5.34) * | <0.001 |
| Age (years) | 1.04 (1.03, 1.04) * | <0.001 |
| Sex (Male vs Female) | 2.33 (2.08, 2.62) * | <0.001 |
| Marital status (In a current marriage vs Single) | 0.84 (0.71, 1.01) | 0.060 |
| Marital status (Divorced or widowed vs Single) | 0.63 (0.37, 1.08) | 0.091 |
| Education level (College or undergraduate vs High school or below) | 0.80 (0.68, 0.94) * | 0.006 |
| Education level (Postgraduate vs High school or below) | 0.66 (0.52, 0.82) * | <0.001 |
| Model 3 (Model 2 + BMI) |  |  |
| O_3_ (Q2 vs Q1) | 2.01 (1.56, 2.60) * | <0.001 |
| O_3_ (Q3 vs Q1) | 2.31 (1.63, 3.28) * | <0.001 |
| O_3_ (Q4 vs Q1) | 3.76 (2.78, 5.09) * | <0.001 |
| Age (years) | 1.04 (1.03, 1.04) * | <0.001 |
| Sex (Male vs Female) | 1.93 (1.71, 2.17) * | <0.001 |
| Marital status (In a current marriage vs Single) | 0.80 (0.67, 0.95) * | 0.013 |
| Marital status (Divorced or widowed vs Single) | 0.62 (0.36, 1.07) | 0.086 |
| Education level (College or undergraduate vs High school or below) | 0.82 (0.69, 0.96) * | 0.016 |
| Education level (Postgraduate vs High school or below) | 0.70 (0.56, 0.87) * | 0.002 |
| BMI (kg/m^2^) | 1.10 (1.09, 1.12) * | <0.001 |
| Model 4 (Model 3 + Family history) |  |  |
| O_3_ (Q2 vs Q1) | 2.00 (1.55, 2.58) * | <0.001 |
| O_3_ (Q3 vs Q1) | 2.30 (1.62, 3.27) * | <0.001 |
| O_3_ (Q4 vs Q1) | 3.76 (2.78, 5.09) * | <0.001 |
| Age(years) | 1.04 (1.03, 1.04) * | <0.001 |
| Sex (Male vs Female) | 1.95 (1.73, 2.20) * | <0.001 |
| Marital status (In a current marriage vs Single) | 0.79 (0.66, 0.95) * | 0.012 |
| Marital status (Divorced or widowed vs Single) | 0.62 (0.36, 1.06) | 0.080 |
| Education level (College or undergraduate vs High school or below) | 0.81 (0.69, 0.96) * | 0.013 |
| Education level (Postgraduate vs High school or below) | 0.69 (0.55, 0.87) * | 0.001 |
| BMI (kg/m^2^) | 1.10 (1.09, 1.12) * | <0.001 |
| Family history of hypertension (Positive vs Negative) | 1.16 (1.04, 1.29) * | 0.010 |
| Family history of hypertension (Unknown vs Negative) | 1.01 (0.78, 1.30) | 0.964 |
| Model 5 (Model 4 + Indoor air pollution + Lifestyle factors) |  |  |
| O_3_ (Q2 vs Q1) | 1.85 (1.40, 2.43) * | <0.001 |
| O_3_ (Q3 vs Q1) | 2.21 (1.52, 3.19) * | <0.001 |
| O_3_ (Q4 vs Q1) | 3.65 (2.64, 5.06) * | <0.001 |
| Age (years) | 1.04 (1.03, 1.04) * | <0.001 |
| Sex (Male vs Female) | 1.99 (1.73, 2.29) * | <0.001 |
| Marital status (In a current marriage vs Single) | 0.81 (0.66, 0.98) * | 0.034 |
| Marital status (Divorced or widowed vs Single) | 0.50 (0.27, 0.95) * | 0.033 |
| Education level (College or undergraduate vs High school or below) | 0.83 (0.69, 0.98) * | 0.030 |
| Education level (Postgraduate vs High school or below) | 0.68 (0.54, 0.87) * | 0.002 |
| BMI (kg/m^2^) | 1.10 (1.08, 1.12) * | <0.001 |
| Family history of hypertension (Positive vs Negative) | 1.18 (1.05, 1.33) * | 0.006 |
| Family history of hypertension (Unknown vs Negative) | 1.04 (0.79, 1.36) | 0.781 |
| Daily cooking time (0–1 hour vs 0 hours) | 0.94 (0.81, 1.08) | 0.382 |
| Daily cooking time (>1 hour vs 0 hours) | 0.91 (0.77, 1.08) | 0.275 |
| Night sleep duration (<7 hours/day vs 7–8 hours/day) | 0.85 (0.69, 1.05) | 0.126 |
| Night sleep duration (>8 hours/day vs 7–8 hours/day) | 0.96 (0.81, 1.13) | 0.601 |
| Smoking (Current vs Never) | 0.92 (0.79, 1.07) | 0.276 |
| Smoking (Former vs Never) | 1.07 (0.79, 1.44) | 0.664 |
| Alcohol drinking (Current vs Never) | 1.09 (0.95, 1.24) | 0.235 |
| Alcohol drinking (Former vs Never) | 0.93 (0.60, 1.43) | 0.729 |
| Physical exercise (Yes vs No) | 1.07 (0.94, 1.20) | 0.310 |
| Model 6 (Model 5 + Personal protective measures against air pollution) |  |  |
| O_3_ (Q2 vs Q1) | 1.85 (1.40, 2.43) * | <0.001 |
| O_3_ (Q3 vs Q1) | 2.20 (1.52, 3.18) * | <0.001 |
| O_3_ (Q4 vs Q1) | 3.61 (2.61, 5.00) * | <0.001 |
| Age (years) | 1.04 (1.03, 1.04) * | <0.001 |
| Sex (Male vs Female) | 1.97 (1.71, 2.27) * | <0.001 |
| Marital status (In a current marriage vs Single) | 0.81 (0.67, 0.99) * | 0.037 |
| Marital status (Divorced or widowed vs Single) | 0.51 (0.27, 0.96) * | 0.036 |
| Education level (College or undergraduate vs High school or below) | 0.83 (0.70, 0.99) * | 0.034 |
| Education level (Postgraduate vs High school or below) | 0.69 (0.54, 0.88) * | 0.003 |
| BMI (kg/m^2^) | 1.10 (1.08, 1.12) * | <0.001 |
| Family history of hypertension (Positive vs Negative) | 1.18 (1.05, 1.33) * | 0.006 |
| Family history of hypertension (Unknown vs Negative) | 1.04 (0.79, 1.36) | 0.785 |
| Daily cooking time (0–1 hour vs 0 hours) | 0.94 (0.82, 1.09) | 0.414 |
| Daily cooking time (>1 hour vs 0 hours) | 0.92 (0.78, 1.09) | 0.322 |
| Night sleep duration (<7 hours/day vs 7–8 hours/day) | 0.85 (0.69, 1.05) | 0.123 |
| Night sleep duration (>8 hours/day vs 7–8 hours/day) | 0.96 (0.81, 1.13) | 0.595 |
| Smoking (Current vs Never) | 0.92 (0.79, 1.07) | 0.271 |
| Smoking (Former vs Never) | 1.07 (0.79, 1.44) | 0.672 |
| Alcohol drinking (Current vs Never) | 1.09 (0.95, 1.24) | 0.230 |
| Alcohol drinking (Former vs Never) | 0.93 (0.60, 1.43) | 0.732 |
| Physical exercise (Yes vs No) | 1.07 (0.95, 1.21) | 0.282 |
| Mask usage (Yes vs No) | 0.96 (0.85, 1.10) | 0.594 |
| Air purifier usage (Yes vs No) | 0.96 (0.84, 1.10) | 0.597 |
| Model 7 (Model 6 + Biochemical indicators and chronic diseases) |  |  |
| O_3_ (Q2 vs Q1) | 1.77 (1.34, 2.36) * | <0.001 |
| O_3_ (Q3 vs Q1) | 2.06 (1.42, 3.00) * | <0.001 |
| O_3_ (Q4 vs Q1) | 3.43 (2.46, 4.79) * | <0.001 |
| Age (years) | 1.03 (1.03, 1.04) * | <0.001 |
| Sex (Male vs Female) | 1.88 (1.62, 2.19) * | <0.001 |
| Marital status (In a current marriage vs Single) | 0.81 (0.66, 0.99) * | 0.042 |
| Marital status (Divorced or widowed vs Single) | 0.58 (0.31, 1.09) | 0.093 |
| Education level (College or undergraduate vs High school or below) | 0.84 (0.70, 1.00) | 0.050 |
| Education level (Postgraduate vs High school or below) | 0.69 (0.54, 0.89) * | 0.004 |
| BMI (kg/m^2^) | 1.09 (1.07, 1.11) * | <0.001 |
| Family history of hypertension (Positive vs Negative) | 1.18 (1.05, 1.33) * | 0.007 |
| Family history of hypertension (Unknown vs Negative) | 1.05 (0.80, 1.38) | 0.741 |
| Daily cooking time (0–1 hour vs 0 hours) | 0.95 (0.82, 1.10) | 0.521 |
| Daily cooking time (>1 hour vs 0 hours) | 0.93 (0.78, 1.11) | 0.414 |
| Night sleep duration (<7 hours/day vs 7–8 hours/day) | 0.86 (0.70, 1.06) | 0.162 |
| Night sleep duration (>8 hours/day vs 7–8 hours/day) | 0.96 (0.81, 1.14) | 0.639 |
| Smoking (Current vs Never) | 0.90 (0.78, 1.05) | 0.186 |
| Smoking (Former vs Never) | 1.10 (0.82, 1.48) | 0.532 |
| Alcohol drinking (Current vs Never) | 1.07 (0.93, 1.23) | 0.335 |
| Alcohol drinking (Former vs Never) | 0.91 (0.58, 1.41) | 0.662 |
| Physical exercise (Yes vs No) | 1.08 (0.95, 1.22) | 0.249 |
| Mask usage (Yes vs No) | 0.95 (0.83, 1.08) | 0.437 |
| Air purifier usage (Yes vs No) | 0.96 (0.84, 1.10) | 0.569 |
| FBG (mmol/L) | 1.04 (1.00, 1.09) | 0.051 |
| TG (mmol/L) | 1.01 (0.95, 1.07) | 0.776 |
| TC (mmol/L) | 1.15 (0.99, 1.33) | 0.063 |
| LDL-C (mmol/L) | 0.98 (0.83, 1.16) | 0.814 |
| HDL-C (mmol/L) | 0.76 (0.58, 0.99) * | 0.042 |
| CHD (Yes vs No) | 0.81 (0.44, 1.50) | 0.507 |
| Cancer (Yes vs No) | 0.53 (0.17, 1.69) | 0.286 |

Note: HR, hazard ratio; CI, confidence interval; O_3_, ozone; BMI, body mass index; FBG, fasting blood glucose; TG, triglyceride; TC, total cholesterol; LDL-C, low-density lipoprotein cholesterol; HDL-C, high-density lipoprotein cholesterol; CHD, coronary heart disease; vs, versus; Q1–Q4, the first to the fourth quartile groups of O_3_ exposure concentrations.

* P-value < 0.05.
