# Supplementary material for: Long-term exposure to ambient ozone at workplace is positively and non-linearly associated with incident hypertension and blood pressure: longitudinal evidence from the Beijing-Tianjin-Hebei medical examination cohort
Source: BMC Public Health. 2023 Oct 16;23:2011. doi: 10.1186/s12889-023-16932-w (PMC10577958; doi:10.1186/s12889-023-16932-w)
Supplement: Supplementary file 2 — Supplementary Material 2 [file 12889_2023_16932_MOESM2_ESM.docx]

**Table S2** Relationship between long-term O_3_ exposure concentrations and DBP derived from the nested mixed-effects linear models

| **Model** | $\boldsymbol{\beta}^{\mathbf{a}}$**(95% CI)** | **P-value** |
| --- | --- | --- |
| Model 1 |  |  |
| O_3_ (Q2 vs Q1) | 1.06 (0.50, 1.62) * | <0.001 |
| O_3_ (Q3 vs Q1) | 0.59 (−0.16, 1.34) | 0.124 |
| O_3_ (Q4 vs Q1) | 0.71 (0.09, 1.34) * | 0.026 |
| Model 2 (Model 1 + Sociodemographic characteristics) |  |  |
| O_3_ (Q2 vs Q1) | 1.02 (0.46, 1.58) * | <0.001 |
| O_3_ (Q3 vs Q1) | 0.59 (−0.16, 1.34) | 0.124 |
| O_3_ (Q4 vs Q1) | 0.70 (0.07, 1.33) * | 0.029 |
| Age (years) | 0.02 (0.01, 0.04) * | 0.007 |
| Sex (Male vs Female) | 0.83 (0.56, 1.10) * | <0.001 |
| Marital status (In a current marriage vs Single) | 0.31 (−0.07, 0.69) | 0.108 |
| Marital status (Divorced or widowed vs Single) | 0.37 (−0.84, 1.59) | 0.547 |
| Education level (College or undergraduate vs High school or below) | −0.16 (−0.61, 0.28) | 0.474 |
| Education level (Postgraduate vs High school or below) | −0.37 (−0.93, 0.18) | 0.187 |
| Model 3 (Model 2 + BMI) |  |  |
| O_3_ (Q2 vs Q1) | 1.03 (0.47, 1.59) * | <0.001 |
| O_3_ (Q3 vs Q1) | 0.55 (−0.20, 1.31) | 0.151 |
| O_3_ (Q4 vs Q1) | 0.67 (0.03, 1.30) * | 0.039 |
| Age (years) | 0.02 (0.00, 0.04) * | 0.011 |
| Sex (Male vs Female) | 0.71 (0.43, 1.00) * | <0.001 |
| Marital status (In a current marriage vs Single) | 0.30 (−0.08, 0.69) | 0.123 |
| Marital status (Divorced or widowed vs Single) | 0.45 (−0.78, 1.68) | 0.471 |
| Education level (College or undergraduate vs High school or below) | −0.16 (−0.61, 0.29) | 0.479 |
| Education level (Postgraduate vs High school or below) | −0.39 (−0.95, 0.17) | 0.170 |
| BMI (kg/m^2^) | 0.06 (0.02, 0.10) * | 0.004 |
| Model 4 (Model 3 + Family history) |  |  |
| O_3_ (Q2 vs Q1) | 1.02 (0.46, 1.59) * | <0.001 |
| O_3_ (Q3 vs Q1) | 0.56 (−0.20, 1.31) | 0.150 |
| O_3_ (Q4 vs Q1) | 0.67 (0.04, 1.31) * | 0.037 |
| Age(years) | 0.02 (0.00, 0.04) * | 0.012 |
| Sex (Male vs Female) | 0.73 (0.44, 1.01) * | <0.001 |
| Marital status (In a current marriage vs Single) | 0.30 (−0.09, 0.68) | 0.130 |
| Marital status (Divorced or widowed vs Single) | 0.44 (−0.78, 1.67) | 0.478 |
| Education level (College or undergraduate vs High school or below) | −0.17 (−0.62, 0.28) | 0.448 |
| Education level (Postgraduate vs High school or below) | −0.40 (−0.96, 0.15) | 0.157 |
| BMI (kg/m^2^) | 0.06 (0.02, 0.10) * | 0.004 |
| Family history of hypertension (Positive vs Negative) | 0.13 (−0.14, 0.41) | 0.351 |
| Family history of hypertension (Unknown vs Negative) | −0.16 (−0.73, 0.40) | 0.568 |
| Model 5 (Model 4 + Indoor air pollution + Lifestyle factors) |  |  |
| O_3_ (Q2 vs Q1) | 0.99 (0.39, 1.59) * | 0.001 |
| O_3_ (Q3 vs Q1) | 0.54 (−0.26, 1.34) | 0.187 |
| O_3_ (Q4 vs Q1) | 0.56 (−0.12, 1.24) | 0.105 |
| Age (years) | 0.02 (0.00, 0.04) * | 0.027 |
| Sex (Male vs Female) | 0.79 (0.44, 1.14) * | <0.001 |
| Marital status (In a current marriage vs Single) | 0.28 (−0.14, 0.70) | 0.194 |
| Marital status (Divorced or widowed vs Single) | −0.05 (−1.38, 1.28) | 0.939 |
| Education level (College or undergraduate vs High school or below) | −0.23 (−0.70, 0.25) | 0.346 |
| Education level (Postgraduate vs High school or below) | −0.55 (−1.14, 0.05) | 0.070 |
| BMI (kg/m^2^) | 0.04 (−0.01, 0.08) | 0.084 |
| Family history of hypertension (Positive vs Negative) | 0.15 (−0.14, 0.45) | 0.309 |
| Family history of hypertension (Unknown vs Negative) | −0.20 (−0.80, 0.39) | 0.505 |
| Daily cooking time (0–1 hour vs 0 hours) | −0.01 (−0.35, 0.32) | 0.935 |
| Daily cooking time (>1 hour vs 0 hours) | 0.16 (−0.25, 0.57) | 0.448 |
| Night sleep duration (<7 hours/day vs 7–8 hours/day) | −0.13 (−0.66, 0.41) | 0.639 |
| Night sleep duration (>8 hours/day vs 7–8 hours/day) | 0.33 (−0.05, 0.72) | 0.092 |
| Smoking (Current vs Never) | −0.15 (−0.59, 0.28) | 0.493 |
| Smoking (Former vs Never) | −0.45 (−1.37, 0.46) | 0.334 |
| Alcohol drinking (Current vs Never) | 0.22 (−0.16, 0.60) | 0.262 |
| Alcohol drinking (Former vs Never) | 0.31 (−1.12, 1.73) | 0.672 |
| Physical exercise (Yes vs No) | −0.15 (−0.46, 0.16) | 0.342 |
| Model 6 (Model 5 + Personal protective measures against air pollution) |  |  |
| O_3_ (Q2 vs Q1) | 0.98 (0.38, 1.59) * | 0.001 |
| O_3_ (Q3 vs Q1) | 0.51 (−0.29, 1.31) | 0.212 |
| O_3_ (Q4 vs Q1) | 0.53 (−0.15, 1.21) | 0.126 |
| Age (years) | 0.02 (0.00, 0.04) * | 0.039 |
| Sex (Male vs Female) | 0.77 (0.42, 1.12) * | <0.001 |
| Marital status (In a current marriage vs Single) | 0.32 (−0.11, 0.74) | 0.142 |
| Marital status (Divorced or widowed vs Single) | −0.02 (−1.35, 1.32) | 0.980 |
| Education level (College or undergraduate vs High school or below) | −0.20 (−0.68, 0.27) | 0.402 |
| Education level (Postgraduate vs High school or below) | −0.51 (−1.10, 0.09) | 0.097 |
| BMI (kg/m^2^) | 0.04 (−0.01, 0.08) | 0.089 |
| Family history of hypertension (Positive vs Negative) | 0.17 (−0.13, 0.46) | 0.267 |
| Family history of hypertension (Unknown vs Negative) | −0.21 (−0.81, 0.39) | 0.488 |
| Daily cooking time (0–1 hour vs 0 hours) | 0.00 (−0.33, 0.34) | 0.986 |
| Daily cooking time (>1 hour vs 0 hours) | 0.18 (−0.23, 0.59) | 0.381 |
| Night sleep duration (<7 hours/day vs 7–8 hours/day) | −0.13 (−0.66, 0.40) | 0.631 |
| Night sleep duration (>8 hours/day vs 7–8 hours/day) | 0.34 (−0.05, 0.72) | 0.089 |
| Smoking (Current vs Never) | −0.15 (−0.59, 0.29) | 0.502 |
| Smoking (Former vs Never) | −0.46 (−1.38, 0.46) | 0.325 |
| Alcohol drinking (Current vs Never) | 0.22 (−0.16, 0.60) | 0.255 |
| Alcohol drinking (Former vs Never) | 0.31 (−1.11, 1.74) | 0.668 |
| Physical exercise (Yes vs No) | −0.14 (−0.45, 0.18) | 0.393 |
| Mask usage (Yes vs No) | −0.04 (−0.36, 0.28) | 0.817 |
| Air purifier usage (Yes vs No) | −0.26 (−0.58, 0.05) | 0.105 |
| Model 7 (Model 6 + Biochemical indicators and chronic diseases) |  |  |
| O_3_ (Q2 vs Q1) | 0.65 (0.01, 1.30) * | 0.047 |
| O_3_ (Q3 vs Q1) | 0.28 (−0.54, 1.10) | 0.502 |
| O_3_ (Q4 vs Q1) | 0.33 (−0.38, 1.03) | 0.369 |
| Age (years) | 0.02 (0.00, 0.04) * | 0.018 |
| Sex (Male vs Female) | 0.67 (0.30, 1.03) * | <0.001 |
| Marital status (In a current marriage vs Single) | 0.30 (−0.14, 0.73) | 0.186 |
| Marital status (Divorced or widowed vs Single) | 0.20 (−1.17, 1.56) | 0.776 |
| Education level (College or undergraduate vs High school or below) | −0.06 (−0.54, 0.42) | 0.809 |
| Education level (Postgraduate vs High school or below) | −0.38 (−0.99, 0.23) | 0.217 |
| BMI (kg/m^2^) | 0.02 (−0.03, 0.07) | 0.391 |
| Family history of hypertension (Positive vs Negative) | 0.17 (−0.13, 0.47) | 0.277 |
| Family history of hypertension (Unknown vs Negative) | −0.12 (−0.73, 0.49) | 0.701 |
| Daily cooking time (0–1 hour vs 0 hours) | −0.04 (−0.38, 0.31) | 0.833 |
| Daily cooking time (>1 hour vs 0 hours) | 0.13 (−0.29, 0.55) | 0.549 |
| Night sleep duration (<7 hours/day vs 7–8 hours/day) | −0.11 (−0.65, 0.44) | 0.705 |
| Night sleep duration (>8 hours/day vs 7–8 hours/day) | 0.31 (−0.09, 0.70) | 0.129 |
| Smoking (Current vs Never) | −0.06 (−0.50, 0.38) | 0.793 |
| Smoking (Former vs Never) | −0.40 (−1.34, 0.53) | 0.396 |
| Alcohol drinking (Current vs Never) | 0.26 (−0.13, 0.64) | 0.190 |
| Alcohol drinking (Former vs Never) | 0.25 (−1.19, 1.70) | 0.732 |
| Physical exercise (Yes vs No) | −0.11 (−0.43, 0.20) | 0.480 |
| Mask usage (Yes vs No) | 0.00 (−0.33, 0.32) | 0.987 |
| Air purifier usage (Yes vs No) | −0.22 (−0.54, 0.10) | 0.180 |
| FBG (mmol/L) | −0.11 (−0.27, 0.04) | 0.140 |
| TG (mmol/L) | −0.02 (−0.20, 0.16) | 0.840 |
| TC (mmol/L) | −0.10 (−0.55, 0.34) | 0.648 |
| LDL-C (mmol/L) | 0.12 (−0.37, 0.61) | 0.634 |
| HDL-C (mmol/L) | −0.67 (−1.34, 0.00) * | 0.050 |
| CHD (Yes vs No) | 0.32 (−1.53, 2.18) | 0.731 |
| Cancer (Yes vs No) | −0.05 (−2.13, 2.02) | 0.960 |

Note: CI, confidence interval; O_3_, ozone; BMI, body mass index; FBG, fasting blood glucose; TG, triglyceride; TC, total cholesterol; LDL-C, low-density lipoprotein cholesterol; HDL-C, high-density lipoprotein cholesterol; CHD, coronary heart disease; vs, versus; Q1–Q4, the first to the fourth quartile groups of O_3_ exposure concentrations.

^a^$\beta$ represents the average increase in the outcomes compared to Q1.

* P-value < 0.05.
