# Supplementary material for: Long-term exposure to ambient ozone at workplace is positively and non-linearly associated with incident hypertension and blood pressure: longitudinal evidence from the Beijing-Tianjin-Hebei medical examination cohort
Source: BMC Public Health. 2023 Oct 16;23:2011. doi: 10.1186/s12889-023-16932-w (PMC10577958; doi:10.1186/s12889-023-16932-w)
Supplement: Supplementary file 3 — Supplementary Material 3 [file 12889_2023_16932_MOESM3_ESM.docx]

**Table S3** Relationship between long-term O_3_ exposure concentrations and SBP derived from the nested mixed-effects linear models

| **Model** | $\boldsymbol{\beta}^{\mathbf{a}}$**(95% CI)** | **P-value** |
| --- | --- | --- |
| Model 1 |  |  |
| O_3_ (Q2 vs Q1) | 3.77 (2.99, 4.55) * | <0.001 |
| O_3_ (Q3 vs Q1) | 3.11 (2.07, 4.15) * | <0.001 |
| O_3_ (Q4 vs Q1) | 3.30 (2.43, 4.17) * | <0.001 |
| Model 2 (Model 1 + Sociodemographic characteristics) |  |  |
| O_3_ (Q2 vs Q1) | 3.72 (2.94, 4.50) * | <0.001 |
| O_3_ (Q3 vs Q1) | 3.13 (2.09, 4.16) * | <0.001 |
| O_3_ (Q4 vs Q1) | 3.34 (2.47, 4.21) * | <0.001 |
| Age (years) | 0.09 (0.07, 0.11) * | <0.001 |
| Sex (Male vs Female) | 0.26 (−0.12, 0.63) | 0.183 |
| Marital status (In a current marriage vs Single) | 0.35 (−0.19, 0.89) | 0.201 |
| Marital status (Divorced or widowed vs Single) | 0.33 (−1.38, 2.05) | 0.704 |
| Education level (College or undergraduate vs High school or below) | −0.49 (−1.12, 0.14) | 0.124 |
| Education level (Postgraduate vs High school or below) | −0.89 (−1.67, −0.11) * | 0.025 |
| Model 3 (Model 2 + BMI) |  |  |
| O_3_ (Q2 vs Q1) | 3.60 (2.82, 4.38) * | <0.001 |
| O_3_ (Q3 vs Q1) | 2.99 (1.95, 4.02) * | <0.001 |
| O_3_ (Q4 vs Q1) | 3.23 (2.36, 4.10) * | <0.001 |
| Age (years) | 0.09 (0.06, 0.11) * | <0.001 |
| Sex (Male vs Female) | 0.04 (−0.36, 0.44) | 0.846 |
| Marital status (In a current marriage vs Single) | 0.31 (−0.23, 0.85) | 0.257 |
| Marital status (Divorced or widowed vs Single) | 0.39 (−1.34, 2.12) | 0.659 |
| Education level (College or undergraduate vs High school or below) | −0.49 (−1.12, 0.14) | 0.128 |
| Education level (Postgraduate vs High school or below) | −0.90 (−1.69, −0.12) * | 0.024 |
| BMI (kg/m^2^) | 0.10 (0.04, 0.15) * | <0.001 |
| Model 4 (Model 3 + Family history) |  |  |
| O_3_ (Q2 vs Q1) | 3.59 (2.81, 4.37) * | <0.001 |
| O_3_ (Q3 vs Q1) | 2.99 (1.96, 4.03) * | <0.001 |
| O_3_ (Q4 vs Q1) | 3.24 (2.37, 4.11) * | <0.001 |
| Age(years) | 0.09 (0.06, 0.11) * | <0.001 |
| Sex (Male vs Female) | 0.08 (−0.32, 0.48) | 0.707 |
| Marital status (In a current marriage vs Single) | 0.30 (−0.24, 0.84) | 0.282 |
| Marital status (Divorced or widowed vs Single) | 0.35 (−1.38, 2.08) | 0.691 |
| Education level (College or undergraduate vs High school or below) | −0.53 (−1.16, 0.10) | 0.100 |
| Education level (Postgraduate vs High school or below) | −0.95 (−1.73, −0.16) * | 0.018 |
| BMI (kg/m^2^) | 0.09 (0.04, 0.15) * | <0.001 |
| Family history of hypertension (Positive vs Negative) | 0.46 (0.07, 0.84) * | 0.021 |
| Family history of hypertension (Unknown vs Negative) | −0.05 (−0.84, 0.74) | 0.907 |
| Model 5 (Model 4 + Indoor air pollution + Lifestyle factors) |  |  |
| O_3_ (Q2 vs Q1) | 3.53 (2.70, 4.36) * | <0.001 |
| O_3_ (Q3 vs Q1) | 3.06 (1.97, 4.15) * | <0.001 |
| O_3_ (Q4 vs Q1) | 3.20 (2.27, 4.13) * | <0.001 |
| Age (years) | 0.08 (0.06, 0.10) * | <0.001 |
| Sex (Male vs Female) | 0.34 (−0.15, 0.84) | 0.172 |
| Marital status (In a current marriage vs Single) | 0.45 (−0.15, 1.04) | 0.142 |
| Marital status (Divorced or widowed vs Single) | −0.19 (−2.08, 1.70) | 0.843 |
| Education level (College or undergraduate vs High school or below) | −0.72 (−1.39, −0.05) * | 0.034 |
| Education level (Postgraduate vs High school or below) | −1.15 (−1.99, −0.32) * | 0.007 |
| BMI (kg/m^2^) | 0.07 (0.01, 0.14) * | 0.015 |
| Family history of hypertension (Positive vs Negative) | 0.53 (0.11, 0.94) * | 0.013 |
| Family history of hypertension (Unknown vs Negative) | −0.01 (−0.86, 0.83) | 0.978 |
| Daily cooking time (0–1 hour vs 0 hours) | −0.07 (−0.55, 0.41) | 0.779 |
| Daily cooking time (>1 hour vs 0 hours) | −0.08 (−0.66, 0.49) | 0.774 |
| Night sleep duration (<7 hours/day vs 7–8 hours/day) | 0.15 (−0.61, 0.90) | 0.706 |
| Night sleep duration (>8 hours/day vs 7–8 hours/day) | 0.00 (−0.54, 0.55) | 0.986 |
| Smoking (Current vs Never) | −0.44 (−1.05, 0.18) | 0.166 |
| Smoking (Former vs Never) | −1.56 (−2.86, −0.26) * | 0.018 |
| Alcohol drinking (Current vs Never) | 0.33 (−0.21, 0.86) | 0.237 |
| Alcohol drinking (Former vs Never) | 0.99 (−1.03, 3.01) | 0.337 |
| Physical exercise (Yes vs No) | −0.35 (−0.79, 0.09) | 0.122 |
| Model 6 (Model 5 + Personal protective measures against air pollution) |  |  |
| O_3_ (Q2 vs Q1) | 3.53 (2.70, 4.36) * | <0.001 |
| O_3_ (Q3 vs Q1) | 3.05 (1.95, 4.14) * | <0.001 |
| O_3_ (Q4 vs Q1) | 3.17 (2.24, 4.11) * | <0.001 |
| Age (years) | 0.08 (0.05, 0.10) * | <0.001 |
| Sex (Male vs Female) | 0.28 (−0.21, 0.78) | 0.264 |
| Marital status (In a current marriage vs Single) | 0.52 (−0.08, 1.12) | 0.092 |
| Marital status (Divorced or widowed vs Single) | −0.13 (−2.01, 1.76) | 0.897 |
| Education level (College or undergraduate vs High school or below) | −0.68 (−1.35, −0.01) * | 0.046 |
| Education level (Postgraduate vs High school or below) | −1.08 (−1.92, −0.24) * | 0.012 |
| BMI (kg/m^2^) | 0.07 (0.01, 0.13) * | 0.017 |
| Family history of hypertension (Positive vs Negative) | 0.55 (0.13, 0.96) * | 0.010 |
| Family history of hypertension (Unknown vs Negative) | −0.05 (−0.89, 0.80) | 0.914 |
| Daily cooking time (0–1 hour vs 0 hours) | −0.04 (−0.52, 0.44) | 0.868 |
| Daily cooking time (>1 hour vs 0 hours) | −0.04 (−0.62, 0.54) | 0.905 |
| Night sleep duration (<7 hours/day vs 7–8 hours/day) | 0.14 (−0.62, 0.89) | 0.726 |
| Night sleep duration (>8 hours/day vs 7–8 hours/day) | 0.01 (−0.54, 0.56) | 0.974 |
| Smoking (Current vs Never) | −0.45 (−1.07, 0.17) | 0.156 |
| Smoking (Former vs Never) | −1.58 (−2.88, −0.28) * | 0.017 |
| Alcohol drinking (Current vs Never) | 0.32 (−0.22, 0.86) | 0.248 |
| Alcohol drinking (Former vs Never) | 0.98 (−1.04, 3.00) | 0.340 |
| Physical exercise (Yes vs No) | −0.31 (−0.75, 0.13) | 0.167 |
| Mask usage (Yes vs No) | −0.35 (−0.80, 0.10) | 0.125 |
| Air purifier usage (Yes vs No) | −0.41 (−0.85, 0.04) | 0.073 |
| Model 7 (Model 6 + Biochemical indicators and chronic diseases) |  |  |
| O_3_ (Q2 vs Q1) | 2.88 (2.00, 3.77) * | <0.001 |
| O_3_ (Q3 vs Q1) | 2.49 (1.36, 3.61) * | <0.001 |
| O_3_ (Q4 vs Q1) | 2.61 (1.64, 3.58) * | <0.001 |
| Age (years) | 0.07 (0.05, 0.10) * | <0.001 |
| Sex (Male vs Female) | 0.15 (−0.37, 0.67) | 0.575 |
| Marital status (In a current marriage vs Single) | 0.66 (0.04, 1.28) * | 0.038 |
| Marital status (Divorced or widowed vs Single) | 0.17 (−1.77, 2.11) | 0.863 |
| Education level (College or undergraduate vs High school or below) | −0.50 (−1.18, 0.19) | 0.153 |
| Education level (Postgraduate vs High school or below) | −0.91 (−1.77, −0.05) * | 0.038 |
| BMI (kg/m^2^) | 0.03 (−0.04, 0.10) | 0.364 |
| Family history of hypertension (Positive vs Negative) | 0.54 (0.12, 0.97) * | 0.013 |
| Family history of hypertension (Unknown vs Negative) | 0.02 (−0.85, 0.89) | 0.961 |
| Daily cooking time (0–1 hour vs 0 hours) | −0.04 (−0.53, 0.45) | 0.875 |
| Daily cooking time (>1 hour vs 0 hours) | −0.06 (−0.66, 0.53) | 0.841 |
| Night sleep duration (<7 hours/day vs 7–8 hours/day) | 0.28 (−0.49, 1.05) | 0.479 |
| Night sleep duration (>8 hours/day vs 7–8 hours/day) | 0.01 (−0.55, 0.58) | 0.959 |
| Smoking (Current vs Never) | −0.41 (−1.04, 0.23) | 0.208 |
| Smoking (Former vs Never) | −1.44 (−2.76, −0.12) * | 0.033 |
| Alcohol drinking (Current vs Never) | 0.33 (−0.22, 0.88) | 0.239 |
| Alcohol drinking (Former vs Never) | 0.70 (−1.35, 2.75) | 0.505 |
| Physical exercise (Yes vs No) | −0.24 (−0.69, 0.20) | 0.286 |
| Mask usage (Yes vs No) | −0.35 (−0.81, 0.11) | 0.139 |
| Air purifier usage (Yes vs No) | −0.38 (−0.84, 0.07) | 0.101 |
| FBG (mmol/L) | 0.02 (−0.19, 0.24) | 0.847 |
| TG (mmol/L) | −0.06 (−0.31, 0.20) | 0.669 |
| TC (mmol/L) | 0.14 (−0.48, 0.77) | 0.656 |
| LDL-C (mmol/L) | 0.00 (−0.69, 0.69) | 0.992 |
| HDL-C (mmol/L) | −0.95 (−1.90, 0.00) * | 0.049 |
| CHD (Yes vs No) | 1.86 (−0.77, 4.49) | 0.167 |
| Cancer (Yes vs No) | −2.30 (−5.25, 0.64) | 0.125 |

Note: CI, confidence interval; O_3_, ozone; BMI, body mass index; FBG, fasting blood glucose; TG, triglyceride; TC, total cholesterol; LDL-C, low-density lipoprotein cholesterol; HDL-C, high-density lipoprotein cholesterol; CHD, coronary heart disease; vs, versus; Q1–Q4, the first to the fourth quartile groups of O_3_ exposure concentrations.

^a^$\beta$ represents the average increase in the outcomes compared to Q1.

* P-value < 0.05.
