# Supplementary material for: Long-term exposure to ambient ozone at workplace is positively and non-linearly associated with incident hypertension and blood pressure: longitudinal evidence from the Beijing-Tianjin-Hebei medical examination cohort
Source: BMC Public Health. 2023 Oct 16;23:2011. doi: 10.1186/s12889-023-16932-w (PMC10577958; doi:10.1186/s12889-023-16932-w)
Supplement: Supplementary file 4 — Supplementary Material 4 [file 12889_2023_16932_MOESM4_ESM.docx]

**Table S4** Relationship between long-term O_3_ exposure concentrations and PP derived from the nested mixed-effects linear models

| **Model** | $\boldsymbol{\beta}^{\mathbf{a}}$**(95% CI)** | **P-value** |
| --- | --- | --- |
| Model 1 |  |  |
| O_3_ (Q2 vs Q1) | 2.63 (1.97, 3.30) * | <0.001 |
| O_3_ (Q3 vs Q1) | 2.35 (1.56, 3.15) * | <0.001 |
| O_3_ (Q4 vs Q1) | 2.48 (1.79, 3.16) * | <0.001 |
| Model 2 (Model 1 + Sociodemographic characteristics) |  |  |
| O_3_ (Q2 vs Q1) | 2.61 (1.95, 3.28) * | <0.001 |
| O_3_ (Q3 vs Q1) | 2.35 (1.55, 3.14) * | <0.001 |
| O_3_ (Q4 vs Q1) | 2.51 (1.82, 3.19) * | <0.001 |
| Age (years) | 0.07 (0.05, 0.09) * | <0.001 |
| Sex (Male vs Female) | −0.59 (−0.91, −0.27) * | <0.001 |
| Marital status (In a current marriage vs Single) | 0.05 (−0.41, 0.51) | 0.830 |
| Marital status (Divorced or widowed vs Single) | −0.01 (−1.46, 1.45) | 0.993 |
| Education level (College or undergraduate vs High school or below) | −0.29 (−0.83, 0.25) | 0.288 |
| Education level (Postgraduate vs High school or below) | −0.48 (−1.15, 0.18) | 0.156 |
| Model 3 (Model 2 + BMI) |  |  |
| O_3_ (Q2 vs Q1) | 2.50 (1.84, 3.17) * | <0.001 |
| O_3_ (Q3 vs Q1) | 2.29 (1.49, 3.08) * | <0.001 |
| O_3_ (Q4 vs Q1) | 2.46 (1.77, 3.15) * | <0.001 |
| Age (years) | 0.07 (0.05, 0.09) * | <0.001 |
| Sex (Male vs Female) | −0.68 (−1.02, −0.34) * | <0.001 |
| Marital status (In a current marriage vs Single) | 0.02 (−0.44, 0.48) | 0.919 |
| Marital status (Divorced or widowed vs Single) | −0.03 (−1.50, 1.44) | 0.971 |
| Education level (College or undergraduate vs High school or below) | −0.29 (−0.83, 0.25) | 0.286 |
| Education level (Postgraduate vs High school or below) | −0.49 (−1.16, 0.18) | 0.154 |
| BMI (kg/m^2^) | 0.04 (−0.01, 0.08) | 0.127 |
| Model 4 (Model 3 + Family history) |  |  |
| O_3_ (Q2 vs Q1) | 2.50 (1.83, 3.16) * | <0.001 |
| O_3_ (Q3 vs Q1) | 2.31 (1.51, 3.10) * | <0.001 |
| O_3_ (Q4 vs Q1) | 2.48 (1.79, 3.16) * | <0.001 |
| Age(years) | 0.07 (0.05, 0.09) * | <0.001 |
| Sex (Male vs Female) | −0.66 (−1.00, −0.32) * | <0.001 |
| Marital status (In a current marriage vs Single) | 0.01 (−0.45, 0.47) | 0.960 |
| Marital status (Divorced or widowed vs Single) | −0.06 (−1.53, 1.41) | 0.935 |
| Education level (College or undergraduate vs High school or below) | −0.32 (−0.86, 0.22) | 0.242 |
| Education level (Postgraduate vs High school or below) | −0.52 (−1.19, 0.15) | 0.129 |
| BMI (kg/m^2^) | 0.04 (−0.01, 0.08) | 0.148 |
| Family history of hypertension (Positive vs Negative) | 0.34 (0.01, 0.67) * | 0.041 |
| Family history of hypertension (Unknown vs Negative) | 0.12 (−0.56, 0.79) | 0.736 |
| Model 5 (Model 4 + Indoor air pollution + Lifestyle factors) |  |  |
| O_3_ (Q2 vs Q1) | 2.44 (1.74, 3.15) * | <0.001 |
| O_3_ (Q3 vs Q1) | 2.29 (1.46, 3.11) * | <0.001 |
| O_3_ (Q4 vs Q1) | 2.47 (1.74, 3.20) * | <0.001 |
| Age (years) | 0.06 (0.04, 0.08) * | <0.001 |
| Sex (Male vs Female) | −0.45 (−0.87, −0.03) * | 0.034 |
| Marital status (In a current marriage vs Single) | 0.19 (−0.32, 0.69) | 0.465 |
| Marital status (Divorced or widowed vs Single) | −0.08 (−1.68, 1.51) | 0.917 |
| Education level (College or undergraduate vs High school or below) | −0.45 (−1.01, 0.12) | 0.122 |
| Education level (Postgraduate vs High school or below) | −0.56 (−1.28, 0.15) | 0.121 |
| BMI (kg/m^2^) | 0.04 (−0.02, 0.09) | 0.174 |
| Family history of hypertension (Positive vs Negative) | 0.40 (0.04, 0.75) * | 0.028 |
| Family history of hypertension (Unknown vs Negative) | 0.18 (−0.53, 0.89) | 0.614 |
| Daily cooking time (0–1 hour vs 0 hours) | −0.06 (−0.46, 0.34) | 0.778 |
| Daily cooking time (>1 hour vs 0 hours) | −0.25 (−0.74, 0.23) | 0.309 |
| Night sleep duration (<7 hours/day vs 7–8 hours/day) | 0.29 (−0.35, 0.92) | 0.378 |
| Night sleep duration (>8 hours/day vs 7–8 hours/day) | −0.34 (−0.80, 0.12) | 0.149 |
| Smoking (Current vs Never) | −0.28 (−0.80, 0.24) | 0.289 |
| Smoking (Former vs Never) | −1.11 (−2.21, −0.02) * | 0.047 |
| Alcohol drinking (Current vs Never) | 0.10 (−0.36, 0.55) | 0.671 |
| Alcohol drinking (Former vs Never) | 0.69 (−1.02, 2.39) | 0.430 |
| Physical exercise (Yes vs No) | −0.19 (−0.56, 0.18) | 0.314 |
| Model 6 (Model 5 + Personal protective measures against air pollution) |  |  |
| O_3_ (Q2 vs Q1) | 2.47 (1.76, 3.17) * | <0.001 |
| O_3_ (Q3 vs Q1) | 2.31 (1.48, 3.14) * | <0.001 |
| O_3_ (Q4 vs Q1) | 2.48 (1.74, 3.21) * | <0.001 |
| Age (years) | 0.06 (0.04, 0.08) * | <0.001 |
| Sex (Male vs Female) | −0.49 (−0.91, −0.07) * | 0.023 |
| Marital status (In a current marriage vs Single) | 0.22 (−0.29, 0.72) | 0.399 |
| Marital status (Divorced or widowed vs Single) | −0.06 (−1.65, 1.54) | 0.945 |
| Education level (College or undergraduate vs High school or below) | −0.43 (−1.00, 0.13) | 0.133 |
| Education level (Postgraduate vs High school or below) | −0.54 (−1.26, 0.17) | 0.138 |
| BMI (kg/m^2^) | 0.03 (−0.02, 0.09) | 0.186 |
| Family history of hypertension (Positive vs Negative) | 0.40 (0.05, 0.75) * | 0.026 |
| Family history of hypertension (Unknown vs Negative) | 0.16 (−0.55, 0.87) | 0.665 |
| Daily cooking time (0–1 hour vs 0 hours) | −0.04 (−0.44, 0.36) | 0.840 |
| Daily cooking time (>1 hour vs 0 hours) | −0.22 (−0.71, 0.27) | 0.373 |
| Night sleep duration (<7 hours/day vs 7–8 hours/day) | 0.28 (−0.36, 0.92) | 0.391 |
| Night sleep duration (>8 hours/day vs 7–8 hours/day) | −0.34 (−0.80, 0.12) | 0.151 |
| Smoking (Current vs Never) | −0.30 (−0.82, 0.23) | 0.267 |
| Smoking (Former vs Never) | −1.12 (−2.22, −0.03) * | 0.045 |
| Alcohol drinking (Current vs Never) | 0.09 (−0.37, 0.54) | 0.704 |
| Alcohol drinking (Former vs Never) | 0.68 (−1.03, 2.38) | 0.437 |
| Physical exercise (Yes vs No) | −0.17 (−0.54, 0.20) | 0.379 |
| Mask usage (Yes vs No) | −0.30 (−0.68, 0.08) | 0.125 |
| Air purifier usage (Yes vs No) | −0.15 (−0.52, 0.23) | 0.441 |
| Model 7 (Model 6 + Biochemical indicators and chronic diseases) |  |  |
| O_3_ (Q2 vs Q1) | 2.12 (1.36, 2.87) * | <0.001 |
| O_3_ (Q3 vs Q1) | 2.03 (1.18, 2.87) * | <0.001 |
| O_3_ (Q4 vs Q1) | 2.14 (1.38, 2.90) * | <0.001 |
| Age (years) | 0.05 (0.03, 0.07) * | <0.001 |
| Sex (Male vs Female) | −0.52 (−0.97, −0.08) * | 0.020 |
| Marital status (In a current marriage vs Single) | 0.38 (−0.15, 0.90) | 0.160 |
| Marital status (Divorced or widowed vs Single) | 0.01 (−1.62, 1.65) | 0.988 |
| Education level (College or undergraduate vs High school or below) | −0.40 (−0.98, 0.18) | 0.173 |
| Education level (Postgraduate vs High school or below) | −0.50 (−1.23, 0.23) | 0.180 |
| BMI (kg/m^2^) | 0.01 (−0.05, 0.06) | 0.783 |
| Family history of hypertension (Positive vs Negative) | 0.39 (0.03, 0.75) * | 0.032 |
| Family history of hypertension (Unknown vs Negative) | 0.14 (−0.59, 0.88) | 0.698 |
| Daily cooking time (0–1 hour vs 0 hours) | 0.00 (−0.41, 0.41) | 0.991 |
| Daily cooking time (>1 hour vs 0 hours) | −0.20 (−0.70, 0.30) | 0.437 |
| Night sleep duration (<7 hours/day vs 7–8 hours/day) | 0.40 (−0.25, 1.05) | 0.229 |
| Night sleep duration (>8 hours/day vs 7–8 hours/day) | −0.30 (−0.78, 0.17) | 0.214 |
| Smoking (Current vs Never) | −0.35 (−0.88, 0.19) | 0.202 |
| Smoking (Former vs Never) | −1.04 (−2.16, 0.08) | 0.068 |
| Alcohol drinking (Current vs Never) | 0.07 (−0.40, 0.53) | 0.774 |
| Alcohol drinking (Former vs Never) | 0.45 (−1.28, 2.18) | 0.611 |
| Physical exercise (Yes vs No) | −0.12 (−0.50, 0.25) | 0.525 |
| Mask usage (Yes vs No) | −0.33 (−0.72, 0.06) | 0.100 |
| Air purifier usage (Yes vs No) | −0.16 (−0.54, 0.22) | 0.409 |
| FBG (mmol/L) | 0.14 (−0.05, 0.32) | 0.141 |
| TG (mmol/L) | −0.04 (−0.26, 0.18) | 0.741 |
| TC (mmol/L) | 0.22 (−0.31, 0.76) | 0.409 |
| LDL-C (mmol/L) | −0.10 (−0.68, 0.49) | 0.752 |
| HDL-C (mmol/L) | −0.29 (−1.10, 0.51) | 0.475 |
| CHD (Yes vs No) | 1.49 (−0.73, 3.71) | 0.187 |
| Cancer (Yes vs No) | −2.23 (−4.71, 0.26) | 0.079 |

Note: CI, confidence interval; O_3_, ozone; BMI, body mass index; FBG, fasting blood glucose; TG, triglyceride; TC, total cholesterol; LDL-C, low-density lipoprotein cholesterol; HDL-C, high-density lipoprotein cholesterol; CHD, coronary heart disease; vs, versus; Q1–Q4, the first to the fourth quartile groups of O_3_ exposure concentrations.

^a^$\beta$ represents the average increase in the outcomes compared to Q1.

* P-value < 0.05.
