# Supplementary material for: Long-term exposure to ambient ozone at workplace is positively and non-linearly associated with incident hypertension and blood pressure: longitudinal evidence from the Beijing-Tianjin-Hebei medical examination cohort
Source: BMC Public Health. 2023 Oct 16;23:2011. doi: 10.1186/s12889-023-16932-w (PMC10577958; doi:10.1186/s12889-023-16932-w)
Supplement: Supplementary file 5 — Supplementary Material 5 [file 12889_2023_16932_MOESM5_ESM.docx]

**Table S5** Relationship between long-term O_3_ exposure concentrations and MAP derived from the nested mixed-effects linear models

| **Model** | $\boldsymbol{\beta}^{\mathbf{a}}$**(95% CI)** | **P-value** |
| --- | --- | --- |
| Model 1 |  |  |
| O_3_ (Q2 vs Q1) | 1.95 (1.40, 2.50) * | <0.001 |
| O_3_ (Q3 vs Q1) | 1.46 (0.72, 2.19) * | <0.001 |
| O_3_ (Q4 vs Q1) | 1.60 (0.98, 2.22) * | <0.001 |
| Model 2 (Model 1 + Sociodemographic characteristics) |  |  |
| O_3_ (Q2 vs Q1) | 1.90 (1.35, 2.45) * | <0.001 |
| O_3_ (Q3 vs Q1) | 1.45 (0.72, 2.18) * | <0.001 |
| O_3_ (Q4 vs Q1) | 1.60 (0.98, 2.22) * | <0.001 |
| Age (years) | 0.04 (0.03, 0.06) * | <0.001 |
| Sex (Male vs Female) | 0.63 (0.36, 0.90) * | <0.001 |
| Marital status (In a current marriage vs Single) | 0.32 (−0.07, 0.70) | 0.106 |
| Marital status (Divorced or widowed vs Single) | 0.34 (−0.88, 1.57) | 0.584 |
| Education level (College or undergraduate vs High school or below) | −0.28 (−0.73, 0.17) | 0.223 |
| Education level (Postgraduate vs High school or below) | −0.54 (−1.10, 0.01) | 0.056 |
| Model 3 (Model 2 + BMI) |  |  |
| O_3_ (Q2 vs Q1) | 1.87 (1.31, 2.42) * | <0.001 |
| O_3_ (Q3 vs Q1) | 1.38 (0.64, 2.11) * | <0.001 |
| O_3_ (Q4 vs Q1) | 1.54 (0.92, 2.16) * | <0.001 |
| Age (years) | 0.04 (0.03, 0.06) * | <0.001 |
| Sex (Male vs Female) | 0.48 (0.20, 0.77) * | <0.001 |
| Marital status (In a current marriage vs Single) | 0.30 (−0.09, 0.68) | 0.132 |
| Marital status (Divorced or widowed vs Single) | 0.41 (−0.82, 1.65) | 0.511 |
| Education level (College or undergraduate vs High school or below) | −0.28 (−0.73, 0.17) | 0.230 |
| Education level (Postgraduate vs High school or below) | −0.56 (−1.11, 0.00) | 0.051 |
| BMI (kg/m^2^) | 0.07 (0.03, 0.11) * | <0.001 |
| Model 4 (Model 3 + Family history) |  |  |
| O_3_ (Q2 vs Q1) | 1.86 (1.30, 2.41) * | <0.001 |
| O_3_ (Q3 vs Q1) | 1.38 (0.64, 2.12) * | <0.001 |
| O_3_ (Q4 vs Q1) | 1.55 (0.92, 2.17) * | <0.001 |
| Age(years) | 0.04 (0.03, 0.06) * | <0.001 |
| Sex (Male vs Female) | 0.50 (0.22, 0.79) * | <0.001 |
| Marital status (In a current marriage vs Single) | 0.29 (−0.10, 0.67) | 0.144 |
| Marital status (Divorced or widowed vs Single) | 0.40 (−0.84, 1.63) | 0.529 |
| Education level (College or undergraduate vs High school or below) | −0.30 (−0.75, 0.15) | 0.197 |
| Education level (Postgraduate vs High school or below) | −0.58 (−1.14, −0.02) * | 0.042 |
| BMI (kg/m^2^) | 0.07 (0.03, 0.11) * | <0.001 |
| Family history of hypertension (Positive vs Negative) | 0.24 (−0.04, 0.51) | 0.094 |
| Family history of hypertension (Unknown vs Negative) | −0.12 (−0.69, 0.44) | 0.666 |
| Model 5 (Model 4 + Indoor air pollution + Lifestyle factors) |  |  |
| O_3_ (Q2 vs Q1) | 1.81 (1.23, 2.40) * | <0.001 |
| O_3_ (Q3 vs Q1) | 1.39 (0.61, 2.17) * | <0.001 |
| O_3_ (Q4 vs Q1) | 1.46 (0.79, 2.12) * | <0.001 |
| Age (years) | 0.04 (0.02, 0.06) * | <0.001 |
| Sex (Male vs Female) | 0.63 (0.28, 0.98) * | <0.001 |
| Marital status (In a current marriage vs Single) | 0.33 (−0.10, 0.75) | 0.133 |
| Marital status (Divorced or widowed vs Single) | −0.11 (−1.45, 1.23) | 0.875 |
| Education level (College or undergraduate vs High school or below) | −0.40 (−0.87, 0.08) | 0.101 |
| Education level (Postgraduate vs High school or below) | −0.74 (−1.34, −0.15) * | 0.014 |
| BMI (kg/m^2^) | 0.05 (0.01, 0.09) * | 0.023 |
| Family history of hypertension (Positive vs Negative) | 0.27 (−0.02, 0.57) | 0.070 |
| Family history of hypertension (Unknown vs Negative) | −0.14 (−0.74, 0.46) | 0.641 |
| Daily cooking time (0–1 hour vs 0 hours) | −0.03 (−0.37, 0.31) | 0.854 |
| Daily cooking time (>1 hour vs 0 hours) | 0.08 (−0.34, 0.49) | 0.718 |
| Night sleep duration (<7 hours/day vs 7–8 hours/day) | −0.04 (−0.58, 0.50) | 0.883 |
| Night sleep duration (>8 hours/day vs 7–8 hours/day) | 0.22 (−0.17, 0.62) | 0.259 |
| Smoking (Current vs Never) | −0.25 (−0.69, 0.19) | 0.266 |
| Smoking (Former vs Never) | −0.81 (−1.73, 0.12) | 0.088 |
| Alcohol drinking (Current vs Never) | 0.26 (−0.12, 0.64) | 0.185 |
| Alcohol drinking (Former vs Never) | 0.55 (−0.89, 1.98) | 0.457 |
| Physical exercise (Yes vs No) | −0.22 (−0.53, 0.10) | 0.173 |
| Model 6 (Model 5 + Personal protective measures against air pollution) |  |  |
| O_3_ (Q2 vs Q1) | 1.81 (1.22, 2.40) * | <0.001 |
| O_3_ (Q3 vs Q1) | 1.36 (0.58, 2.14) * | <0.001 |
| O_3_ (Q4 vs Q1) | 1.43 (0.76, 2.09) * | <0.001 |
| Age (years) | 0.04 (0.02, 0.06) * | <0.001 |
| Sex (Male vs Female) | 0.60 (0.24, 0.95) * | <0.001 |
| Marital status (In a current marriage vs Single) | 0.37 (−0.05, 0.80) | 0.086 |
| Marital status (Divorced or widowed vs Single) | −0.06 (−1.41, 1.28) | 0.927 |
| Education level (College or undergraduate vs High school or below) | −0.37 (−0.84, 0.11) | 0.131 |
| Education level (Postgraduate vs High school or below) | −0.69 (−1.29, −0.09) * | 0.023 |
| BMI (kg/m^2^) | 0.05 (0.01, 0.09) * | 0.025 |
| Family history of hypertension (Positive vs Negative) | 0.29 (−0.01, 0.59) | 0.056 |
| Family history of hypertension (Unknown vs Negative) | −0.16 (−0.76, 0.44) | 0.602 |
| Daily cooking time (0–1 hour vs 0 hours) | −0.01 (−0.35, 0.33) | 0.947 |
| Daily cooking time (>1 hour vs 0 hours) | 0.11 (−0.30, 0.52) | 0.607 |
| Night sleep duration (<7 hours/day vs 7–8 hours/day) | −0.05 (−0.58, 0.49) | 0.867 |
| Night sleep duration (>8 hours/day vs 7–8 hours/day) | 0.23 (−0.16, 0.62) | 0.252 |
| Smoking (Current vs Never) | −0.25 (−0.69, 0.19) | 0.263 |
| Smoking (Former vs Never) | −0.82 (−1.74, 0.11) | 0.083 |
| Alcohol drinking (Current vs Never) | 0.26 (−0.12, 0.64) | 0.186 |
| Alcohol drinking (Former vs Never) | 0.55 (−0.89, 1.98) | 0.457 |
| Physical exercise (Yes vs No) | −0.20 (−0.51, 0.12) | 0.222 |
| Mask usage (Yes vs No) | −0.14 (−0.46, 0.18) | 0.382 |
| Air purifier usage (Yes vs No) | −0.31 (−0.63, 0.01) | 0.058 |
| Model 7 (Model 6 + Biochemical indicators and chronic diseases) |  |  |
| O_3_ (Q2 vs Q1) | 1.39 (0.76, 2.02) * | <0.001 |
| O_3_ (Q3 vs Q1) | 1.04 (0.24, 1.84) * | 0.011 |
| O_3_ (Q4 vs Q1) | 1.12 (0.43, 1.82) * | 0.002 |
| Age (years) | 0.04 (0.02, 0.06) * | <0.001 |
| Sex (Male vs Female) | 0.48 (0.11, 0.85) * | 0.011 |
| Marital status (In a current marriage vs Single) | 0.41 (−0.04, 0.85) | 0.071 |
| Marital status (Divorced or widowed vs Single) | 0.18 (−1.20, 1.56) | 0.795 |
| Education level (College or undergraduate vs High school or below) | −0.21 (−0.70, 0.28) | 0.398 |
| Education level (Postgraduate vs High school or below) | −0.55 (−1.17, 0.06) | 0.076 |
| BMI (kg/m^2^) | 0.02 (−0.02, 0.07) | 0.328 |
| Family history of hypertension (Positive vs Negative) | 0.29 (−0.02, 0.59) | 0.063 |
| Family history of hypertension (Unknown vs Negative) | −0.08 (−0.69, 0.54) | 0.811 |
| Daily cooking time (0–1 hour vs 0 hours) | −0.04 (−0.39, 0.31) | 0.835 |
| Daily cooking time (>1 hour vs 0 hours) | 0.06 (−0.36, 0.49) | 0.767 |
| Night sleep duration (<7 hours/day vs 7–8 hours/day) | 0.02 (−0.53, 0.57) | 0.947 |
| Night sleep duration (>8 hours/day vs 7–8 hours/day) | 0.21 (−0.19, 0.61) | 0.303 |
| Smoking (Current vs Never) | −0.18 (−0.63, 0.27) | 0.439 |
| Smoking (Former vs Never) | −0.73 (−1.68, 0.21) | 0.126 |
| Alcohol drinking (Current vs Never) | 0.29 (−0.10, 0.68) | 0.149 |
| Alcohol drinking (Former vs Never) | 0.41 (−1.05, 1.87) | 0.580 |
| Physical exercise (Yes vs No) | −0.16 (−0.48, 0.16) | 0.327 |
| Mask usage (Yes vs No) | −0.12 (−0.45, 0.21) | 0.479 |
| Air purifier usage (Yes vs No) | −0.27 (−0.59, 0.05) | 0.100 |
| FBG (mmol/L) | −0.07 (−0.22, 0.08) | 0.362 |
| TG (mmol/L) | −0.02 (−0.21, 0.16) | 0.796 |
| TC (mmol/L) | −0.05 (−0.50, 0.39) | 0.810 |
| LDL-C (mmol/L) | 0.12 (−0.37, 0.61) | 0.639 |
| HDL-C (mmol/L) | −0.75 (−1.43, −0.08) * | 0.029 |
| CHD (Yes vs No) | 0.82 (−1.05, 2.69) | 0.392 |
| Cancer (Yes vs No) | −0.79 (−2.88, 1.31) | 0.461 |

Note: CI, confidence interval; O_3_, ozone; BMI, body mass index; FBG, fasting blood glucose; TG, triglyceride; TC, total cholesterol; LDL-C, low-density lipoprotein cholesterol; HDL-C, high-density lipoprotein cholesterol; CHD, coronary heart disease; vs, versus; Q1–Q4, the first to the fourth quartile groups of O_3_ exposure concentrations.

^a^$\beta$ represents the average increase in the outcomes compared to Q1.

* P-value < 0.05.
