# Supplementary material for: Long-term exposure to ambient ozone at workplace is positively and non-linearly associated with incident hypertension and blood pressure: longitudinal evidence from the Beijing-Tianjin-Hebei medical examination cohort
Source: BMC Public Health. 2023 Oct 16;23:2011. doi: 10.1186/s12889-023-16932-w (PMC10577958; doi:10.1186/s12889-023-16932-w)
Supplement: Supplementary file 6 — Supplementary Material 6 [file 12889_2023_16932_MOESM6_ESM.docx]

**Table S6** *P* value for interaction terms of subgroup in the full models based on five outcomes

| **Interaction items** | | **Hypertension** | **DBP** | **SBP** | **PP** | **MAP** |
| --- | --- | --- | --- | --- | --- | --- |
| Sex | Q1 | – | – | – | – | – |
|  | Q2 | 0.047 * | <0.001 * | 0.056 | 0.191 | <0.001 * |
|  | Q3 | 0.596 | 0.728 | 0.487 | 0.227 | 0.932 |
|  | Q4 | 0.595 | 0.147 | 0.933 | 0.241 | 0.307 |
| Age | Q1 | – | – | – | – | – |
|  | Q2 | 0.056 | 0.244 | 0.010 * | 0.038 * | 0.049 * |
|  | Q3 | 0.246 | 0.862 | 0.018 * | 0.008 * | 0.217 |
|  | Q4 | 0.692 | 0.945 | 0.047 * | 0.018 * | 0.381 |
| BMI | Q1 | – | – | – | – | – |
|  | Q2 | 0.026 * | 0.542 | 0.261 | 0.454 | 0.343 |
|  | Q3 | 0.025 * | 0.093 | 0.515 | 0.578 | 0.158 |
|  | Q4 | 0.194 | 0.011 * | 0.438 | 0.270 | 0.040 * |

Note: DBP, diastolic blood pressure; SBP, systolic blood pressure; PP, pulse pressure; MAP, mean arterial pressure; BMI, body mass index; Q1–Q4, the first to the fourth quartile groups of O_3_ exposure concentrations.

* P-value < 0.05.
