# Supplementary material for: Long-term exposure to ambient ozone at workplace is positively and non-linearly associated with incident hypertension and blood pressure: longitudinal evidence from the Beijing-Tianjin-Hebei medical examination cohort
Source: BMC Public Health. 2023 Oct 16;23:2011. doi: 10.1186/s12889-023-16932-w (PMC10577958; doi:10.1186/s12889-023-16932-w)
Supplement: Supplementary file 7 — Supplementary Material 7 [file 12889_2023_16932_MOESM7_ESM.docx]

**Table S7** Results of the full nested mixed-effects model analysis after excluding 98 participants with self-reported hypertension

| **Variables** | **HR/**$\boldsymbol{\beta}^{\mathbf{a}}$**(95% CI)** | **P-value** |
| --- | --- | --- |
| Outcome of hypertension |  |  |
| O_3_ (Q2 vs Q1) | 1.79 (1.34, 2.40) * | <0.001 |
| O_3_ (Q3 vs Q1) | 2.11 (1.43, 3.11) * | <0.001 |
| O_3_ (Q4 vs Q1) | 3.60 (2.56, 5.07) * | <0.001 |
| Age (years) | 1.03 (1.02, 1.04) * | <0.001 |
| Sex (Male vs Female) | 1.85 (1.59, 2.16) * | <0.001 |
| Marital status (In a current marriage vs Single) | 0.80 (0.65, 0.99) * | 0.037 |
| Marital status (Divorced or widowed vs Single) | 0.49 (0.25, 0.99) * | 0.047 |
| Education level (College or undergraduate vs High school or below) | 0.84 (0.70, 1.01) | 0.064 |
| Education level (Postgraduate vs High school or below) | 0.71 (0.55, 0.92) * | 0.009 |
| BMI (kg/m^2^) | 1.09 (1.07, 1.11) * | <0.001 |
| Family history of hypertension (Positive vs Negative) | 1.17 (1.03, 1.32) * | 0.015 |
| Family history of hypertension (Unknown vs Negative) | 1.05 (0.80, 1.38) | 0.718 |
| Daily cooking time (0–1 hour vs 0 hours) | 0.94 (0.81, 1.10) | 0.448 |
| Daily cooking time (>1 hour vs 0 hours) | 0.93 (0.78, 1.10) | 0.392 |
| Night sleep duration (<7 hours/day vs 7–8 hours/day) | 0.86 (0.69, 1.06) | 0.155 |
| Night sleep duration (>8 hours/day vs 7–8 hours/day) | 0.98 (0.82, 1.17) | 0.818 |
| Smoking (Current vs Never) | 0.90 (0.77, 1.05) | 0.186 |
| Smoking (Former vs Never) | 1.10 (0.81, 1.49) | 0.556 |
| Alcohol drinking (Current vs Never) | 1.09 (0.95, 1.25) | 0.237 |
| Alcohol drinking (Former vs Never) | 0.92 (0.59, 1.44) | 0.720 |
| Physical exercise (Yes vs No) | 1.08 (0.95, 1.23) | 0.217 |
| Mask usage (Yes vs No) | 0.93 (0.81, 1.07) | 0.296 |
| Air purifier usage (Yes vs No) | 0.97 (0.84, 1.11) | 0.657 |
| FBG (mmol/L) | 1.05 (1.00, 1.09) * | 0.029 |
| TG (mmol/L) | 1.01 (0.95, 1.07) | 0.786 |
| TC (mmol/L) | 1.15 (0.99, 1.33) | 0.072 |
| LDL-C (mmol/L) | 0.98 (0.82, 1.16) | 0.804 |
| HDL-C (mmol/L) | 0.73 (0.56, 0.96) * | 0.026 |
| CHD (Yes vs No) | 0.83 (0.45, 1.53) | 0.544 |
| Cancer (Yes vs No) | 0.57 (0.18, 1.79) | 0.334 |
| Outcome of DBP |  |  |
| O_3_ (Q2 vs Q1) | 0.68 (0.03, 1.32) * | 0.040 |
| O_3_ (Q3 vs Q1) | 0.20 (−0.63, 1.03) | 0.634 |
| O_3_ (Q4 vs Q1) | 0.33 (−0.38, 1.04) | 0.360 |
| Age (years) | 0.02 (0.00, 0.04) * | 0.024 |
| Sex (Male vs Female) | 0.64 (0.27, 1.01) * | <0.001 |
| Marital status (In a current marriage vs Single) | 0.33 (−0.11, 0.77) | 0.141 |
| Marital status (Divorced or widowed vs Single) | 0.14 (−1.24, 1.51) | 0.846 |
| Education level (College or undergraduate vs High school or below) | −0.06 (−0.54, 0.43) | 0.816 |
| Education level (Postgraduate vs High school or below) | −0.39 (−1.00, 0.22) | 0.210 |
| BMI (kg/m^2^) | 0.02 (−0.03, 0.07) | 0.423 |
| Family history of hypertension (Positive vs Negative) | 0.16 (−0.14, 0.46) | 0.287 |
| Family history of hypertension (Unknown vs Negative) | −0.14 (−0.75, 0.47) | 0.658 |
| Daily cooking time (0–1 hour vs 0 hours) | −0.05 (−0.40, 0.29) | 0.766 |
| Daily cooking time (>1 hour vs 0 hours) | 0.09 (−0.33, 0.51) | 0.671 |
| Night sleep duration (<7 hours/day vs 7–8 hours/day) | −0.10 (−0.64, 0.44) | 0.718 |
| Night sleep duration (>8 hours/day vs 7–8 hours/day) | 0.30 (−0.10, 0.69) | 0.139 |
| Smoking (Current vs Never) | −0.02 (−0.46, 0.43) | 0.944 |
| Smoking (Former vs Never) | −0.35 (−1.29, 0.58) | 0.462 |
| Alcohol drinking (Current vs Never) | 0.25 (−0.14, 0.64) | 0.203 |
| Alcohol drinking (Former vs Never) | 0.24 (−1.20, 1.68) | 0.747 |
| Physical exercise (Yes vs No) | −0.08 (−0.40, 0.23) | 0.603 |
| Mask usage (Yes vs No) | 0.01 (−0.31, 0.34) | 0.929 |
| Air purifier usage (Yes vs No) | −0.25 (−0.57, 0.08) | 0.134 |
| FBG (mmol/L) | −0.11 (−0.26, 0.04) | 0.149 |
| TG (mmol/L) | −0.03 (−0.21, 0.16) | 0.780 |
| TC (mmol/L) | −0.07 (−0.52, 0.37) | 0.755 |
| LDL-C (mmol/L) | 0.07 (−0.42, 0.57) | 0.765 |
| HDL-C (mmol/L) | −0.71 (−1.38, −0.04) * | 0.037 |
| CHD (Yes vs No) | 0.33 (−1.52, 2.18) | 0.729 |
| Cancer (Yes vs No) | −0.02 (−2.09, 2.05) | 0.985 |
| Outcome of SBP |  |  |
| O_3_ (Q2 vs Q1) | 2.97 (2.08, 3.86) * | <0.001 |
| O_3_ (Q3 vs Q1) | 2.36 (1.23, 3.50) * | <0.001 |
| O_3_ (Q4 vs Q1) | 2.61 (1.64, 3.59) * | <0.001 |
| Age (years) | 0.07 (0.05, 0.10) * | <0.001 |
| Sex (Male vs Female) | 0.12 (−0.40, 0.64) | 0.649 |
| Marital status (In a current marriage vs Single) | 0.68 (0.06, 1.30) * | 0.031 |
| Marital status (Divorced or widowed vs Single) | 0.09 (−1.85, 2.04) | 0.925 |
| Education level (College or undergraduate vs High school or below) | −0.44 (−1.13, 0.24) | 0.203 |
| Education level (Postgraduate vs High school or below) | −0.87 (−1.74, −0.01) * | 0.046 |
| BMI (kg/m^2^) | 0.03 (−0.04, 0.10) | 0.383 |
| Family history of hypertension (Positive vs Negative) | 0.54 (0.12, 0.97) * | 0.012 |
| Family history of hypertension (Unknown vs Negative) | 0.05 (−0.82, 0.92) | 0.911 |
| Daily cooking time (0–1 hour vs 0 hours) | −0.03 (−0.52, 0.46) | 0.899 |
| Daily cooking time (>1 hour vs 0 hours) | −0.07 (−0.66, 0.53) | 0.822 |
| Night sleep duration (<7 hours/day vs 7–8 hours/day) | 0.28 (−0.50, 1.05) | 0.485 |
| Night sleep duration (>8 hours/day vs 7–8 hours/day) | 0.00 (−0.57, 0.56) | 0.988 |
| Smoking (Current vs Never) | −0.36 (−0.99, 0.27) | 0.260 |
| Smoking (Former vs Never) | −1.40 (−2.73, −0.08) * | 0.038 |
| Alcohol drinking (Current vs Never) | 0.33 (−0.22, 0.88) | 0.243 |
| Alcohol drinking (Former vs Never) | 0.70 (−1.35, 2.74) | 0.504 |
| Physical exercise (Yes vs No) | −0.22 (−0.67, 0.23) | 0.336 |
| Mask usage (Yes vs No) | −0.34 (−0.80, 0.12) | 0.150 |
| Air purifier usage (Yes vs No) | −0.41 (−0.86, 0.05) | 0.079 |
| FBG (mmol/L) | 0.05 (−0.17, 0.26) | 0.672 |
| TG (mmol/L) | −0.07 (−0.32, 0.19) | 0.612 |
| TC (mmol/L) | 0.20 (−0.42, 0.83) | 0.523 |
| LDL-C (mmol/L) | −0.08 (−0.78, 0.61) | 0.811 |
| HDL-C (mmol/L) | −1.03 (−1.98, −0.08) * | 0.034 |
| CHD (Yes vs No) | 1.89 (−0.73, 4.51) | 0.158 |
| Cancer (Yes vs No) | −2.25 (−5.18, 0.69) | 0.134 |
| Outcome of PP |  |  |
| O_3_ (Q2 vs Q1) | 2.19 (1.44, 2.95) * | <0.001 |
| O_3_ (Q3 vs Q1) | 2.00 (1.15, 2.86) * | <0.001 |
| O_3_ (Q4 vs Q1) | 2.17 (1.41, 2.93) * | <0.001 |
| Age (years) | 0.05 (0.03, 0.07) * | <0.001 |
| Sex (Male vs Female) | −0.53 (−0.97, −0.09) * | 0.019 |
| Marital status (In a current marriage vs Single) | 0.37 (−0.16, 0.89) | 0.167 |
| Marital status (Divorced or widowed vs Single) | 0.00 (−1.65, 1.65) | 1.000 |
| Education level (College or undergraduate vs High school or below) | −0.35 (−0.94, 0.23) | 0.232 |
| Education level (Postgraduate vs High school or below) | −0.46 (−1.19, 0.27) | 0.217 |
| BMI (kg/m^2^) | 0.01 (−0.05, 0.06) | 0.779 |
| Family history of hypertension (Positive vs Negative) | 0.39 (0.03, 0.76) * | 0.032 |
| Family history of hypertension (Unknown vs Negative) | 0.19 (−0.54, 0.93) | 0.603 |
| Daily cooking time (0–1 hour vs 0 hours) | 0.02 (−0.39, 0.43) | 0.920 |
| Daily cooking time (>1 hour vs 0 hours) | −0.17 (−0.67, 0.33) | 0.508 |
| Night sleep duration (<7 hours/day vs 7–8 hours/day) | 0.40 (−0.26, 1.05) | 0.235 |
| Night sleep duration (>8 hours/day vs 7–8 hours/day) | −0.31 (−0.79, 0.16) | 0.200 |
| Smoking (Current vs Never) | −0.35 (−0.88, 0.19) | 0.201 |
| Smoking (Former vs Never) | −1.05 (−2.17, 0.07) | 0.066 |
| Alcohol drinking (Current vs Never) | 0.07 (−0.39, 0.54) | 0.753 |
| Alcohol drinking (Former vs Never) | 0.46 (−1.26, 2.19) | 0.599 |
| Physical exercise (Yes vs No) | −0.13 (−0.50, 0.25) | 0.504 |
| Mask usage (Yes vs No) | −0.33 (−0.72, 0.06) | 0.093 |
| Air purifier usage (Yes vs No) | −0.16 (−0.54, 0.22) | 0.411 |
| FBG (mmol/L) | 0.16 (−0.02, 0.34) | 0.086 |
| TG (mmol/L) | −0.04 (−0.26, 0.18) | 0.735 |
| TC (mmol/L) | 0.25 (−0.28, 0.78) | 0.361 |
| LDL-C (mmol/L) | −0.13 (−0.72, 0.46) | 0.672 |
| HDL-C (mmol/L) | −0.32 (−1.12, 0.49) | 0.438 |
| CHD (Yes vs No) | 1.53 (−0.69, 3.74) | 0.177 |
| Cancer (Yes vs No) | −2.20 (−4.68, 0.28) | 0.082 |
| Outcome of MAP |  |  |
| O_3_ (Q2 vs Q1) | 1.43 (0.80, 2.06) * | <0.001 |
| O_3_ (Q3 vs Q1) | 0.95 (0.15, 1.76) * | 0.021 |
| O_3_ (Q4 vs Q1) | 1.13 (0.44, 1.82) * | 0.001 |
| Age (years) | 0.04 (0.02, 0.06) * | <0.001 |
| Sex (Male vs Female) | 0.46 (0.09, 0.83) * | 0.016 |
| Marital status (In a current marriage vs Single) | 0.44 (0.00, 0.88) | 0.052 |
| Marital status (Divorced or widowed vs Single) | 0.12 (−1.27, 1.50) | 0.870 |
| Education level (College or undergraduate vs High school or below) | −0.19 (−0.68, 0.30) | 0.442 |
| Education level (Postgraduate vs High school or below) | −0.55 (−1.16, 0.07) | 0.081 |
| BMI (kg/m^2^) | 0.02 (−0.02, 0.07) | 0.357 |
| Family history of hypertension (Positive vs Negative) | 0.29 (−0.02, 0.59) | 0.065 |
| Family history of hypertension (Unknown vs Negative) | −0.08 (−0.69, 0.54) | 0.803 |
| Daily cooking time (0–1 hour vs 0 hours) | −0.04 (−0.40, 0.31) | 0.801 |
| Daily cooking time (>1 hour vs 0 hours) | 0.04 (−0.39, 0.46) | 0.865 |
| Night sleep duration (<7 hours/day vs 7–8 hours/day) | 0.02 (−0.53, 0.57) | 0.941 |
| Night sleep duration (>8 hours/day vs 7–8 hours/day) | 0.20 (−0.20, 0.60) | 0.329 |
| Smoking (Current vs Never) | −0.13 (−0.58, 0.32) | 0.560 |
| Smoking (Former vs Never) | −0.69 (−1.63, 0.26) | 0.154 |
| Alcohol drinking (Current vs Never) | 0.28 (−0.11, 0.67) | 0.158 |
| Alcohol drinking (Former vs Never) | 0.40 (−1.05, 1.86) | 0.588 |
| Physical exercise (Yes vs No) | −0.13 (−0.45, 0.19) | 0.420 |
| Mask usage (Yes vs No) | −0.10 (−0.43, 0.22) | 0.534 |
| Air purifier usage (Yes vs No) | −0.30 (−0.62, 0.03) | 0.072 |
| FBG (mmol/L) | −0.06 (−0.21, 0.09) | 0.433 |
| TG (mmol/L) | −0.03 (−0.22, 0.15) | 0.729 |
| TC (mmol/L) | −0.01 (−0.46, 0.43) | 0.953 |
| LDL-C (mmol/L) | 0.06 (−0.43, 0.55) | 0.804 |
| HDL-C (mmol/L) | −0.81 (−1.48, −0.13) * | 0.019 |
| CHD (Yes vs No) | 0.83 (−1.04, 2.70) | 0.383 |
| Cancer (Yes vs No) | −0.75 (−2.83, 1.34) | 0.483 |

Note: HR, hazard ratio; CI, confidence interval; O_3_, ozone; DBP, diastolic blood pressure; SBP, systolic blood pressure; PP, pulse pressure; MAP, mean arterial pressure; BMI, body mass index; FBG, fasting blood glucose; TG, triglyceride; TC, total cholesterol; LDL-C, low-density lipoprotein cholesterol; HDL-C, high-density lipoprotein cholesterol; CHD, coronary heart disease; vs, versus; Q1–Q4, the first to the fourth quartile groups of O_3_ exposure concentrations.

^a^$\beta$ represents the average increase in the outcomes compared to Q1.

* P-value < 0.05.
