# Supplementary material for: Long-term exposure to ambient ozone at workplace is positively and non-linearly associated with incident hypertension and blood pressure: longitudinal evidence from the Beijing-Tianjin-Hebei medical examination cohort
Source: BMC Public Health. 2023 Oct 16;23:2011. doi: 10.1186/s12889-023-16932-w (PMC10577958; doi:10.1186/s12889-023-16932-w)
Supplement: Supplementary file 8 — Supplementary Material 8 [file 12889_2023_16932_MOESM8_ESM.docx]

**Table S8** Results from the full nested mixed-effects model with diabetes and dyslipidemia replacing biochemical indicators

| **Variables** | **HR/**$\boldsymbol{\beta}^{\mathbf{a}}$**(95% CI)** | **P-value** |
| --- | --- | --- |
| Outcome of hypertension |  |  |
| O_3_ (Q2 vs Q1) | 1.73 (1.31, 2.29) * | <0.001 |
| O_3_ (Q3 vs Q1) | 2.10 (1.44, 3.04) * | <0.001 |
| O_3_ (Q4 vs Q1) | 3.41 (2.45, 4.74) * | <0.001 |
| Age (years) | 1.03 (1.03, 1.04) * | <0.001 |
| Sex (Male vs Female) | 1.93 (1.66, 2.23) * | <0.001 |
| Marital status (In a current marriage vs Single) | 0.83 (0.67, 1.01) | 0.064 |
| Marital status (Divorced or widowed vs Single) | 0.54 (0.29, 1.02) | 0.058 |
| Education level (College or undergraduate vs High school or below) | 0.84 (0.70, 0.99) * | 0.044 |
| Education level (Postgraduate vs High school or below) | 0.70 (0.54, 0.89) * | 0.004 |
| BMI (kg/m^2^) | 1.09 (1.07, 1.11) * | <0.001 |
| Family history of hypertension (Positive vs Negative) | 1.19 (1.05, 1.34) * | 0.005 |
| Family history of hypertension (Unknown vs Negative) | 1.07 (0.82, 1.41) | 0.614 |
| Daily cooking time (0–1 hour vs 0 hours) | 0.94 (0.81, 1.08) | 0.372 |
| Daily cooking time (>1 hour vs 0 hours) | 0.93 (0.78, 1.10) | 0.374 |
| Night sleep duration (<7 hours/day vs 7–8 hours/day) | 0.87 (0.70, 1.07) | 0.183 |
| Night sleep duration (>8 hours/day vs 7–8 hours/day) | 0.95 (0.80, 1.13) | 0.592 |
| Smoking (Current vs Never) | 0.91 (0.78, 1.06) | 0.223 |
| Smoking (Former vs Never) | 1.10 (0.82, 1.48) | 0.538 |
| Alcohol drinking (Current vs Never) | 1.06 (0.93, 1.22) | 0.371 |
| Alcohol drinking (Former vs Never) | 0.87 (0.56, 1.36) | 0.551 |
| Physical exercise (Yes vs No) | 1.07 (0.94, 1.21) | 0.290 |
| Mask usage (Yes vs No) | 0.96 (0.84, 1.09) | 0.533 |
| Air purifier usage (Yes vs No) | 0.97 (0.84, 1.11) | 0.638 |
| Diabetes (Yes vs No) | 1.14 (0.91, 1.42) | 0.265 |
| Dyslipidemia (Yes vs No) | 1.25 (1.10, 1.41) * | <0.001 |
| CHD (Yes vs No) | 0.77 (0.42, 1.41) | 0.397 |
| Cancer (Yes vs No) | 0.53 (0.17, 1.67) | 0.276 |
| Outcome of DBP |  |  |
| O_3_ (Q2 vs Q1) | 0.67 (0.03, 1.30) * | 0.041 |
| O_3_ (Q3 vs Q1) | 0.28 (−0.54, 1.10) | 0.508 |
| O_3_ (Q4 vs Q1) | 0.24 (−0.47, 0.94) | 0.507 |
| Age (years) | 0.01 (0.00, 0.03) | 0.107 |
| Sex (Male vs Female) | 0.72 (0.37, 1.08) * | <0.001 |
| Marital status (In a current marriage vs Single) | 0.30 (−0.14, 0.73) | 0.181 |
| Marital status (Divorced or widowed vs Single) | 0.18 (−1.18, 1.54) | 0.799 |
| Education level (College or undergraduate vs High school or below) | −0.14 (−0.62, 0.34) | 0.562 |
| Education level (Postgraduate vs High school or below) | −0.46 (−1.06, 0.14) | 0.135 |
| BMI (kg/m^2^) | 0.03 (−0.01, 0.08) | 0.156 |
| Family history of hypertension (Positive vs Negative) | 0.17 (−0.13, 0.47) | 0.276 |
| Family history of hypertension (Unknown vs Negative) | −0.11 (−0.71, 0.49) | 0.719 |
| Daily cooking time (0–1 hour vs 0 hours) | −0.07 (−0.41, 0.28) | 0.705 |
| Daily cooking time (>1 hour vs 0 hours) | 0.11 (−0.31, 0.53) | 0.606 |
| Night sleep duration (<7 hours/day vs 7–8 hours/day) | −0.10 (−0.64, 0.44) | 0.714 |
| Night sleep duration (>8 hours/day vs 7–8 hours/day) | 0.32 (−0.07, 0.71) | 0.110 |
| Smoking (Current vs Never) | −0.07 (−0.51, 0.38) | 0.770 |
| Smoking (Former vs Never) | −0.40 (−1.32, 0.52) | 0.394 |
| Alcohol drinking (Current vs Never) | 0.19 (−0.19, 0.57) | 0.331 |
| Alcohol drinking (Former vs Never) | 0.24 (−1.20, 1.67) | 0.748 |
| Physical exercise (Yes vs No) | −0.10 (−0.42, 0.21) | 0.516 |
| Mask usage (Yes vs No) | −0.03 (−0.35, 0.29) | 0.865 |
| Air purifier usage (Yes vs No) | −0.25 (−0.56, 0.07) | 0.131 |
| Diabetes (Yes vs No) | −0.37 (−1.14, 0.39) | 0.339 |
| Dyslipidemia (Yes vs No) | 0.36 (0.02, 0.71) * | 0.039 |
| CHD (Yes vs No) | 0.37 (−1.48, 2.22) | 0.692 |
| Cancer (Yes vs No) | −0.10 (−2.17, 1.97) | 0.924 |
| Outcome of SBP |  |  |
| O_3_ (Q2 vs Q1) | 2.90 (2.03, 3.78) * | <0.001 |
| O_3_ (Q3 vs Q1) | 2.56 (1.45, 3.68) * | <0.001 |
| O_3_ (Q4 vs Q1) | 2.60 (1.64, 3.57) * | <0.001 |
| Age (years) | 0.07 (0.04, 0.09) * | <0.001 |
| Sex (Male vs Female) | 0.26 (−0.24, 0.76) | 0.313 |
| Marital status (In a current marriage vs Single) | 0.62 (0.01, 1.24) * | 0.048 |
| Marital status (Divorced or widowed vs Single) | 0.11 (−1.82, 2.04) | 0.912 |
| Education level (College or undergraduate vs High school or below) | −0.58 (−1.25, 0.09) | 0.090 |
| Education level (Postgraduate vs High school or below) | −0.99 (−1.85, −0.14) * | 0.022 |
| BMI (kg/m^2^) | 0.05 (−0.01, 0.11) | 0.129 |
| Family history of hypertension (Positive vs Negative) | 0.55 (0.12, 0.97) * | 0.012 |
| Family history of hypertension (Unknown vs Negative) | 0.04 (−0.81, 0.90) | 0.919 |
| Daily cooking time (0–1 hour vs 0 hours) | −0.08 (−0.57, 0.41) | 0.747 |
| Daily cooking time (>1 hour vs 0 hours) | −0.07 (−0.66, 0.52) | 0.819 |
| Night sleep duration (<7 hours/day vs 7–8 hours/day) | 0.25 (−0.52, 1.02) | 0.522 |
| Night sleep duration (>8 hours/day vs 7–8 hours/day) | 0.04 (−0.52, 0.60) | 0.885 |
| Smoking (Current vs Never) | −0.44 (−1.07, 0.19) | 0.169 |
| Smoking (Former vs Never) | −1.51 (−2.82, −0.21) * | 0.023 |
| Alcohol drinking (Current vs Never) | 0.28 (−0.27, 0.82) | 0.319 |
| Alcohol drinking (Former vs Never) | 0.74 (−1.29, 2.77) | 0.474 |
| Physical exercise (Yes vs No) | −0.28 (−0.72, 0.17) | 0.221 |
| Mask usage (Yes vs No) | −0.37 (−0.82, 0.09) | 0.117 |
| Air purifier usage (Yes vs No) | −0.38 (−0.83, 0.08) | 0.104 |
| Diabetes (Yes vs No) | 0.32 (−0.76, 1.41) | 0.562 |
| Dyslipidemia (Yes vs No) | 0.52 (0.03, 1.01) * | 0.037 |
| CHD (Yes vs No) | 1.81 (−0.82, 4.43) | 0.177 |
| Cancer (Yes vs No) | −2.34 (−5.28, 0.60) | 0.118 |
| Outcome of PP |  |  |
| O_3_ (Q2 vs Q1) | 2.13 (1.38, 2.88) * | <0.001 |
| O_3_ (Q3 vs Q1) | 2.09 (1.24, 2.93) * | <0.001 |
| O_3_ (Q4 vs Q1) | 2.20 (1.45, 2.95) * | <0.001 |
| Age (years) | 0.05 (0.03, 0.08) * | <0.001 |
| Sex (Male vs Female) | −0.47 (−0.89, −0.04) * | 0.032 |
| Marital status (In a current marriage vs Single) | 0.34 (−0.18, 0.86) | 0.196 |
| Marital status (Divorced or widowed vs Single) | −0.02 (−1.65, 1.60) | 0.977 |
| Education level (College or undergraduate vs High school or below) | −0.41 (−0.98, 0.16) | 0.161 |
| Education level (Postgraduate vs High school or below) | −0.51 (−1.24, 0.21) | 0.164 |
| BMI (kg/m^2^) | 0.01 (−0.04, 0.07) | 0.598 |
| Family history of hypertension (Positive vs Negative) | 0.40 (0.04, 0.76) * | 0.029 |
| Family history of hypertension (Unknown vs Negative) | 0.16 (−0.56, 0.88) | 0.669 |
| Daily cooking time (0–1 hour vs 0 hours) | −0.01 (−0.42, 0.40) | 0.967 |
| Daily cooking time (>1 hour vs 0 hours) | −0.18 (−0.67, 0.32) | 0.478 |
| Night sleep duration (<7 hours/day vs 7–8 hours/day) | 0.37 (−0.28, 1.02) | 0.267 |
| Night sleep duration (>8 hours/day vs 7–8 hours/day) | −0.29 (−0.76, 0.18) | 0.230 |
| Smoking (Current vs Never) | −0.37 (−0.90, 0.15) | 0.166 |
| Smoking (Former vs Never) | −1.11 (−2.22, −0.01) * | 0.048 |
| Alcohol drinking (Current vs Never) | 0.08 (−0.38, 0.54) | 0.728 |
| Alcohol drinking (Former vs Never) | 0.51 (−1.20, 2.23) | 0.558 |
| Physical exercise (Yes vs No) | −0.16 (−0.54, 0.21) | 0.392 |
| Mask usage (Yes vs No) | −0.32 (−0.70, 0.07) | 0.106 |
| Air purifier usage (Yes vs No) | −0.13 (−0.51, 0.25) | 0.513 |
| Diabetes (Yes vs No) | 0.70 (−0.21, 1.62) | 0.133 |
| Dyslipidemia (Yes vs No) | 0.17 (−0.24, 0.58) | 0.422 |
| CHD (Yes vs No) | 1.40 (−0.82, 3.61) | 0.217 |
| Cancer (Yes vs No) | −2.23 (−4.71, 0.26) | 0.079 |
| Outcome of MAP |  |  |
| O_3_ (Q2 vs Q1) | 1.40 (0.78, 2.03) * | <0.001 |
| O_3_ (Q3 vs Q1) | 1.06 (0.26, 1.85) * | 0.009 |
| O_3_ (Q4 vs Q1) | 1.06 (0.37, 1.74) * | 0.003 |
| Age (years) | 0.03 (0.02, 0.05) * | <0.001 |
| Sex (Male vs Female) | 0.56 (0.20, 0.92) * | 0.002 |
| Marital status (In a current marriage vs Single) | 0.40 (−0.04, 0.83) | 0.077 |
| Marital status (Divorced or widowed vs Single) | 0.15 (−1.22, 1.52) | 0.833 |
| Education level (College or undergraduate vs High school or below) | −0.29 (−0.77, 0.19) | 0.230 |
| Education level (Postgraduate vs High school or below) | −0.63 (−1.24, −0.03) * | 0.040 |
| BMI (kg/m^2^) | 0.04 (−0.01, 0.08) | 0.100 |
| Family history of hypertension (Positive vs Negative) | 0.29 (−0.01, 0.59) | 0.060 |
| Family history of hypertension (Unknown vs Negative) | −0.06 (−0.67, 0.54) | 0.840 |
| Daily cooking time (0–1 hour vs 0 hours) | −0.07 (−0.42, 0.28) | 0.690 |
| Daily cooking time (>1 hour vs 0 hours) | 0.05 (−0.37, 0.47) | 0.819 |
| Night sleep duration (<7 hours/day vs 7–8 hours/day) | 0.01 (−0.53, 0.56) | 0.966 |
| Night sleep duration (>8 hours/day vs 7–8 hours/day) | 0.23 (−0.17, 0.62) | 0.259 |
| Smoking (Current vs Never) | −0.19 (−0.64, 0.25) | 0.396 |
| Smoking (Former vs Never) | −0.76 (−1.68, 0.17) | 0.111 |
| Alcohol drinking (Current vs Never) | 0.22 (−0.16, 0.61) | 0.257 |
| Alcohol drinking (Former vs Never) | 0.42 (−1.03, 1.86) | 0.573 |
| Physical exercise (Yes vs No) | −0.16 (−0.48, 0.15) | 0.311 |
| Mask usage (Yes vs No) | −0.14 (−0.47, 0.18) | 0.395 |
| Air purifier usage (Yes vs No) | −0.29 (−0.61, 0.03) | 0.080 |
| Diabetes (Yes vs No) | −0.15 (−0.92, 0.62) | 0.706 |
| Dyslipidemia (Yes vs No) | 0.42 (0.07, 0.76) * | 0.018 |
| CHD (Yes vs No) | 0.83 (−1.04, 2.70) | 0.382 |
| Cancer (Yes vs No) | −0.83 (−2.92, 1.26) | 0.435 |

Note: HR, hazard ratio; CI, confidence interval; O_3_, ozone; DBP, diastolic blood pressure; SBP, systolic blood pressure; PP, pulse pressure; MAP, mean arterial pressure; BMI, body mass index; CHD, coronary heart disease; Q1–Q4, the first to the fourth quartile groups of O_3_ exposure concentrations.

^a^$\beta$ represents the average increase in the outcomes compared to Q1.

* P-value < 0.05.
