# Supplementary material for: Long-term exposure to ambient ozone at workplace is positively and non-linearly associated with incident hypertension and blood pressure: longitudinal evidence from the Beijing-Tianjin-Hebei medical examination cohort
Source: BMC Public Health. 2023 Oct 16;23:2011. doi: 10.1186/s12889-023-16932-w (PMC10577958; doi:10.1186/s12889-023-16932-w)
Supplement: Supplementary file 9 — Supplementary Material 9 [file 12889_2023_16932_MOESM9_ESM.docx]

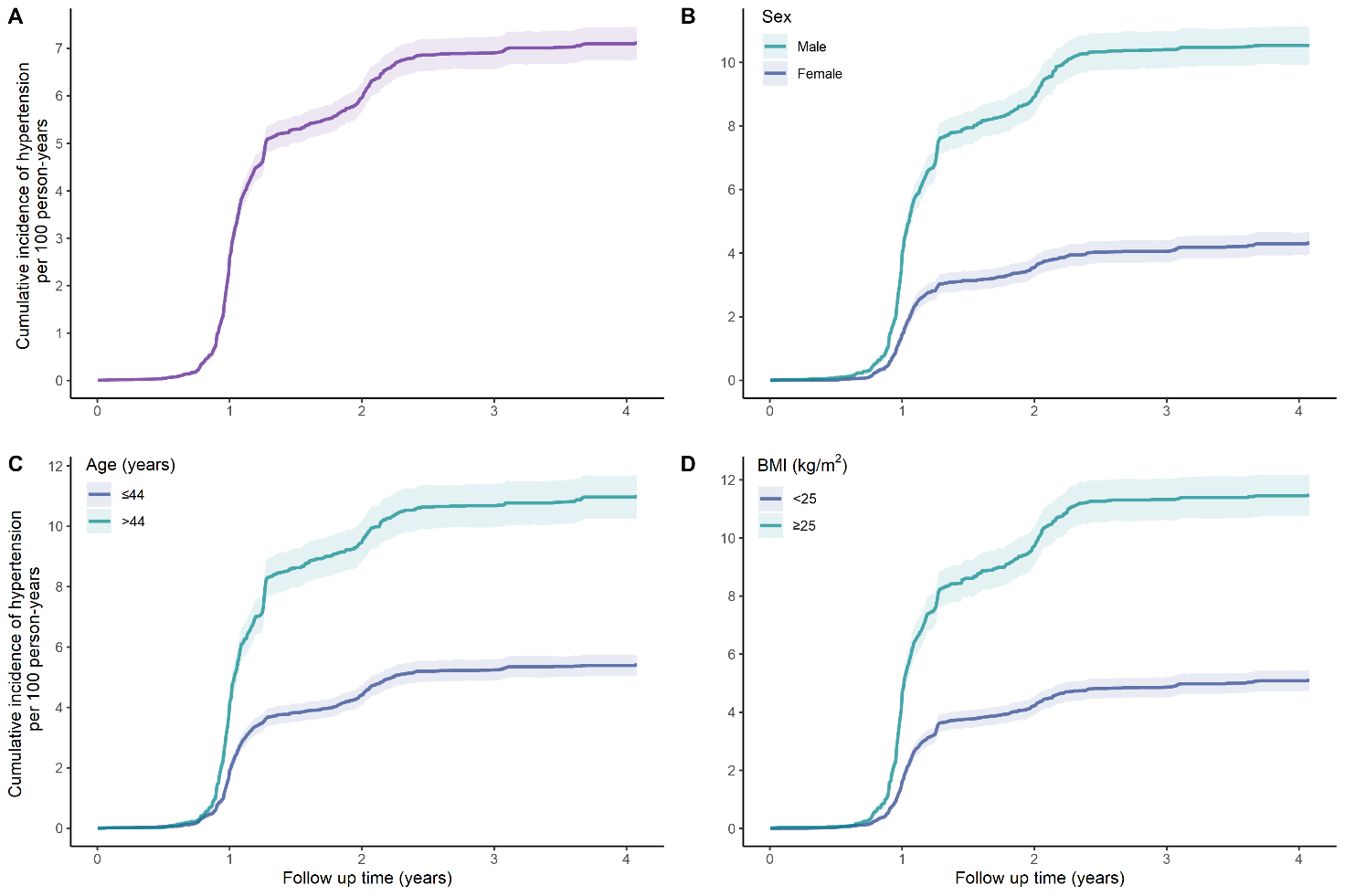


**Fig. S1.** Cumulative incidence of hypertension per 100 person-years during the follow-up period. Panel A shows the overall trend. Panels B, C and D introduce sex (male and female), age (≤44 and >44 years), and BMI (<25 and ≥25 kg/m^2^) as stratified variables, respectively. Shaded areas represent the bias-corrected 95% confidence intervals estimated by repeating the analysis with 1000 bootstrapped samples. Note: BMI, body mass index.
