# Supplementary material for: Long-term exposure to ambient ozone at workplace is positively and non-linearly associated with incident hypertension and blood pressure: longitudinal evidence from the Beijing-Tianjin-Hebei medical examination cohort
Source: BMC Public Health. 2023 Oct 16;23:2011. doi: 10.1186/s12889-023-16932-w (PMC10577958; doi:10.1186/s12889-023-16932-w)
Supplement: Supplementary file 10 — Supplementary Material 10 [file 12889_2023_16932_MOESM10_ESM.docx]

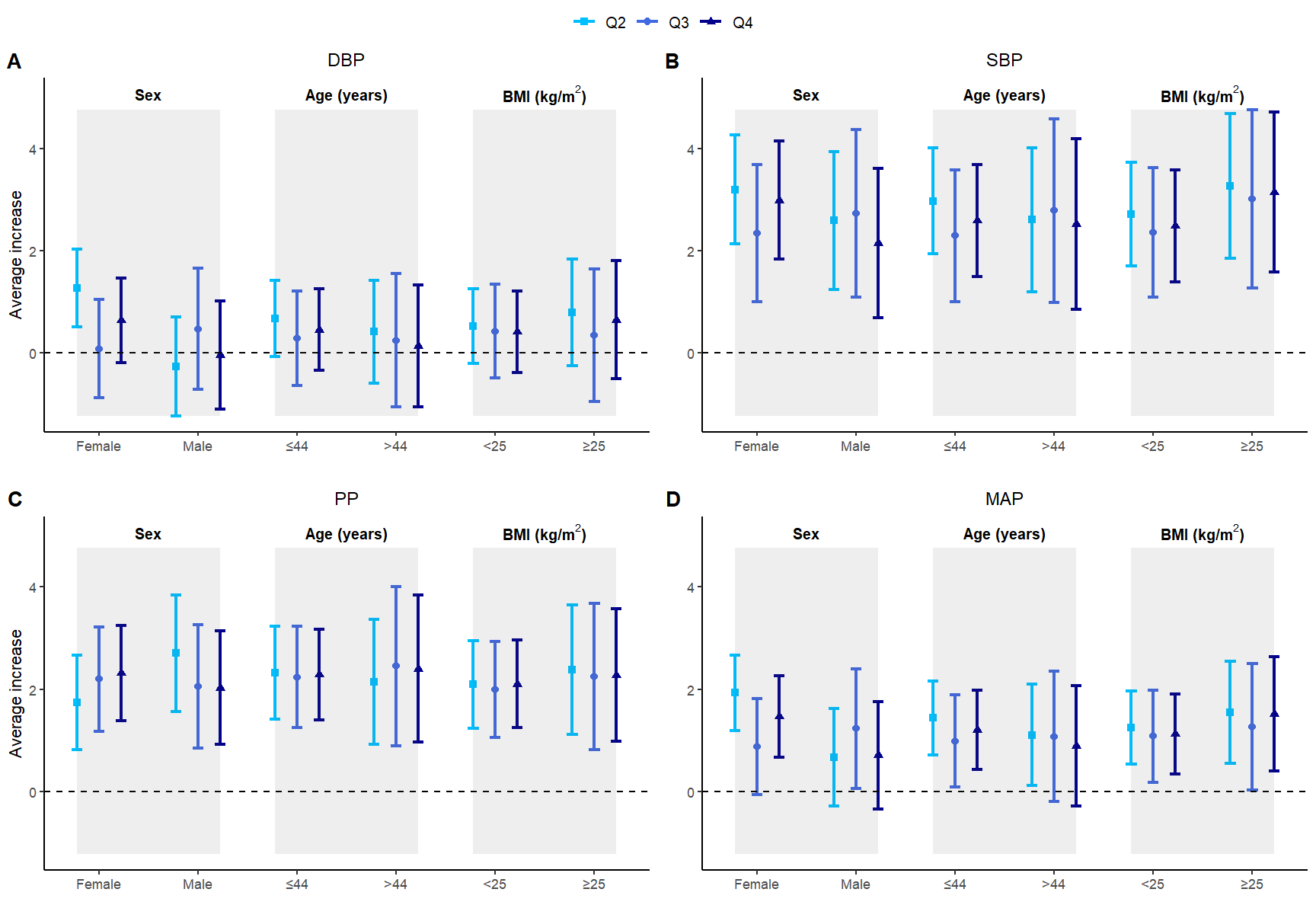


**Fig. S2.** Subgroup effects estimated according to sex (male and female), age (≤44 and >44 years), and BMI (<25 and ≥25 kg/m^2^) for four outcomes based on fully nested mixed-effects linear models. Note: DBP, diastolic blood pressure; SBP, systolic blood pressure; PP, pulse pressure; MAP, mean arterial pressure; BMI, body mass index; Q2–Q4, the second to the fourth quartile groups of O_3_ exposure concentrations.
