# Supplementary material for: Long-term exposure to ambient ozone at workplace is positively and non-linearly associated with incident hypertension and blood pressure: longitudinal evidence from the Beijing-Tianjin-Hebei medical examination cohort
Source: BMC Public Health. 2023 Oct 16;23:2011. doi: 10.1186/s12889-023-16932-w (PMC10577958; doi:10.1186/s12889-023-16932-w)
Supplement: Supplementary file 11 — Supplementary Material 11 [file 12889_2023_16932_MOESM11_ESM.docx]

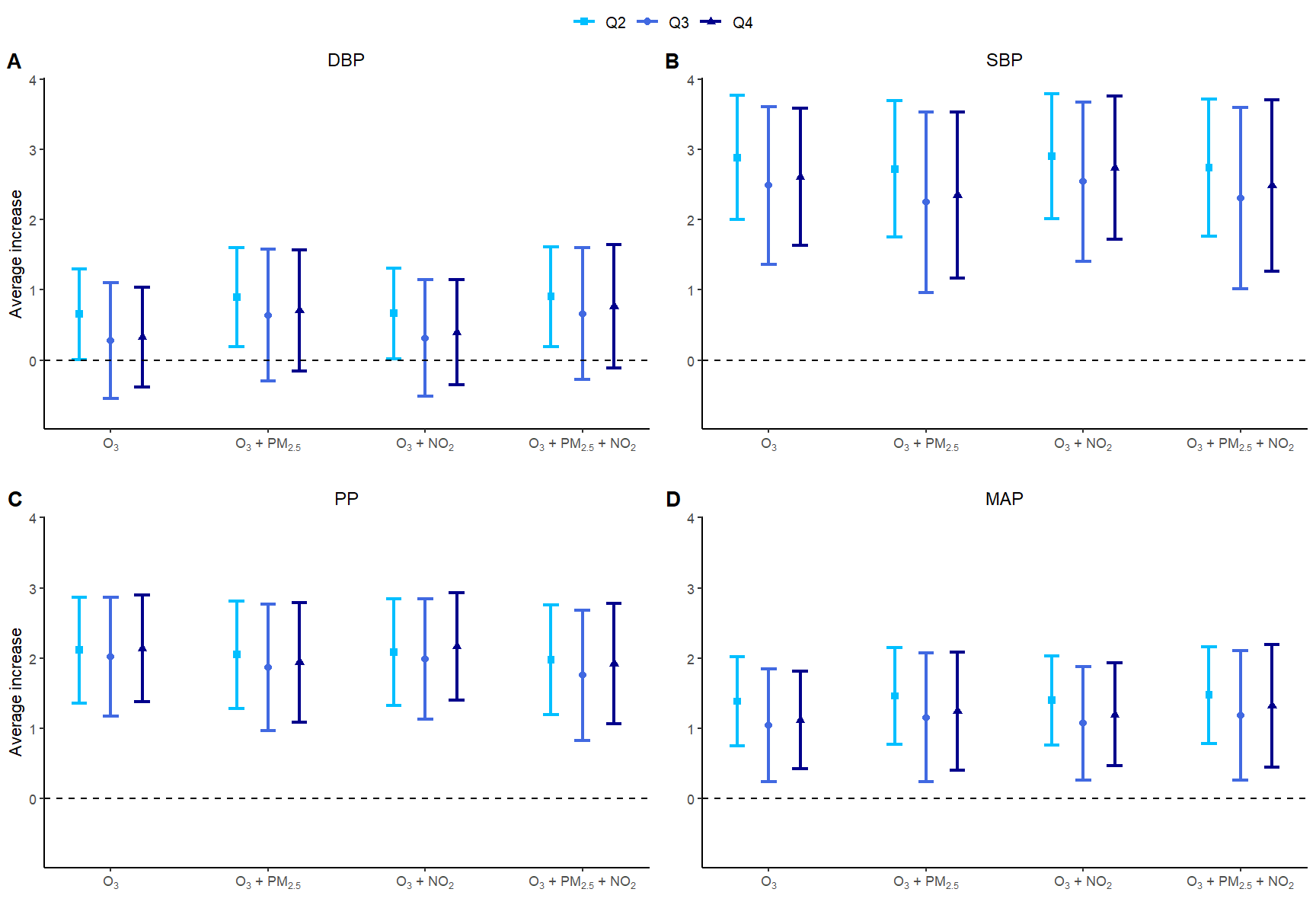


**Fig. S3.** Two-pollutant and three-pollutant models involving the pollutants of PM_2.5_ and NO_2_ for four outcomes based on fully nested mixed-effects linear models. Note: DBP, diastolic blood pressure; SBP, systolic blood pressure; PP, pulse pressure; MAP, mean arterial pressure; O_3_, ozone; PM_2.5_, particulate matter with aerodynamic diameter ≤2.5 μm; NO_2_, nitrogen dioxide; Q2–Q4, the second to the fourth quartile groups of O_3_ exposure concentrations.
