# Supplementary material for: Long-term exposure to ambient ozone at workplace is positively and non-linearly associated with incident hypertension and blood pressure: longitudinal evidence from the Beijing-Tianjin-Hebei medical examination cohort
Source: BMC Public Health. 2023 Oct 16;23:2011. doi: 10.1186/s12889-023-16932-w (PMC10577958; doi:10.1186/s12889-023-16932-w)
Supplement: Supplementary file 13 — Supplementary Material 13 [file 12889_2023_16932_MOESM13_ESM.docx]

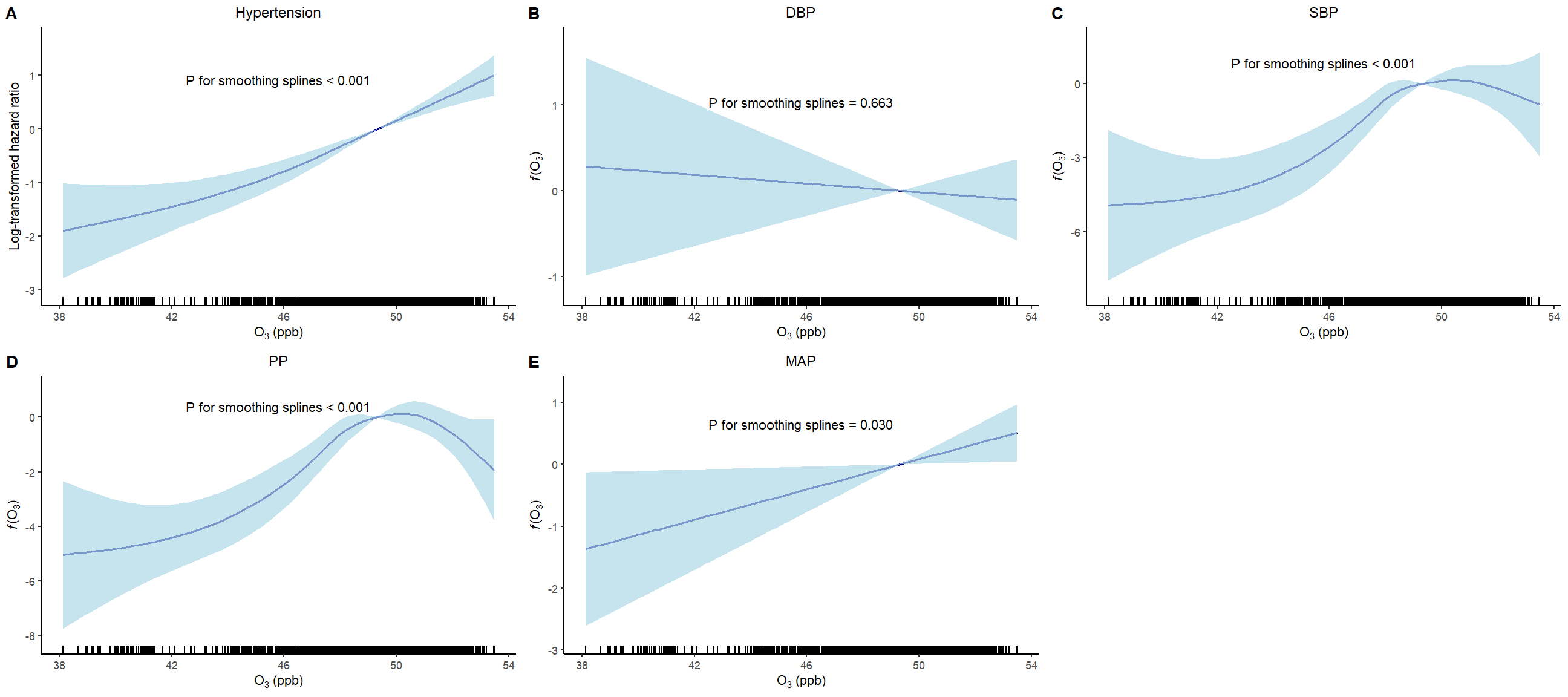


**Fig. S5.** Concentration-response curves of the association of 1-year average O_3_ exposure concentrations with hypertension, DBP, SBP, PP and MAP. Analyses are adjusted for age, sex, marital status, education level, BMI, family history of hypertension, daily cooking time, night sleep duration, smoking, mask usage, air purifier usage, FBG, TG, TC, LDL-C, HDL-C, CHD and cancer. Effects estimates are indicated by solid lines and 95% confidence intervals by shaded areas. Darker colors in the lower bars represent a higher sample clustering. Hazard ratios are on a logarithmic scale. Note: DBP, diastolic blood pressure; SBP, systolic blood pressure; PP, pulse pressure; MAP, mean arterial pressure; O_3_, ozone; ppb, parts per billion.
